# A chromosome-level genome assembly of the *Rhus* gall aphid *Schlechtendalia chinensis* provides insight into the endogenization of *Parvovirus-*like DNA sequences

**DOI:** 10.1186/s12864-023-09916-y

**Published:** 2024-01-02

**Authors:** Aftab Ahmad, Carol von Dohlen, Zhumei Ren

**Affiliations:** 1 School of Life Science, Shanxi University, Taiyuan, Shanxi China; 2 Department of Biology, Utah State University, Logan, Utah United States of America

**Keywords:** *Rhus* gall aphid, *Schlechtendalia chinensis*, Genomics, Endogenization, *Parvovirus*, P450 family

## Abstract

The *Rhus* gall aphid, *Schlechtendalia chinensis*, feeds on its primary host plant *Rhus chinensis* to induce galls, which have economic importance in medicines and the food industry. *Rhus* gall aphids have a unique life cycle and are economically beneficial but there is huge gap in genomic information about this group of aphids. *Schlechtendalia chinensis* induces rich-tannin galls on its host plant and is emerging as a model organism for both commercial applications and applied research in the context of gall production by insects. Here, we generated a high-quality chromosome-level assembly for the *S*. *chinensis* genome, enabling the comparison between *S. chinensis* and non-galling aphids. The final genome assembly is 344.59 Mb with 91.71% of the assembled sequences anchored into 13 chromosomes. We predicted 15,013 genes, of which 14,582 (97.13%) coding genes were annotated, and 99% of the predicted genes were anchored to the 13 chromosomes. This assembly reveals the endogenization of *parvovirus*-related DNA sequences (PRDs) in the *S. chinensis* genome, which could play a role in environmental adaptations. We demonstrated the characterization and classification of cytochrome P450s in the genome assembly, which are functionally crucial for sap-feeding insects and have roles in detoxification and insecticide resistance. This genome assembly also revealed the whole genome duplication events in *S. chinensis*, which can be considered in comparative evolutionary analysis. Our work represents a reference genome for gall-forming aphids that could be used for comparative genomic studies between galling and non-galling aphids and provides the first insight into the endogenization of PRDs in the genome of galling aphids. It also provides novel genetic information for future research on gall-formation and insect-plant interactions.

**Supplementary Information:**

The online version contains supplementary material available at 10.1186/s12864-023-09916-y.

## Introduction

Aphids are well-known phloem-feeding pests of agriculture, reducing yields in some crops by up to 50% [1]. Of the nearly 5,000 known aphid species (Hemiptera: Aphidoidea), among which approximately 450 feed on crop plants, more than 100 are agricultural pests with substantial economic impacts [1–6]. Aside from this minority of pest species, most aphids have little noticeable physical or physiological effects on their host plants. One exception is fewer than 10% of species that induce galls on their host plant. Gall formation is usually associated with life cycles encompassing obligate alternation between a woody (primary) host and a herbaceous (secondary) host. Gall-forming aphids induce galls on the woody host plant and live inside the gall for several generations. The complex life cycles and host specificity of gall-forming aphids make them intriguing models for studying the evolution of insect-plant interactions [7–9].

Because gall-forming insects redirect plant development and resources to form a structure in which they are protected and nourished, the relationship of insect-gall and host plant has long been considered parasitic [10]. Galls may act as nutrient and metabolite sinks, diverting resources away from plant metabolism, and in some cases destroying reproductive structures [11]. In some systems, however, the presence of galls may benefit the host plant [12], or have benign effects [13].

Gall-forming aphids derive mainly from three subfamilies, of which species in the Eriosomatinae, tribe Fordini induce some of the largest and most elaborate gall forms [14]. Within Fordini, *Rhus* gall aphids (Melaphidina) comprise six genera and 13 species and use *Rhus* species as their primary hosts [13, 15]. Unlike other aphids that cause damage to their host plants, this group does not appear to impose any serious fitness costs on its hosts. On the contrary, a recent study demonstrated the occurrence of nutrient exchange between a *Rhus* gall aphid and its host plant, suggesting an association bordering on mutualism [13]. In addition, this study detected no significant increase within gall tissues in the secretion of jasmonic acid [13], which plays a vital role in regulating plant defensive responses against sap-feeding pests. The absence of a defensive response to gall induction further suggests that the relationship between the gall aphid and its host plant is benign [13, 16, 17].

**Fig. 1:**
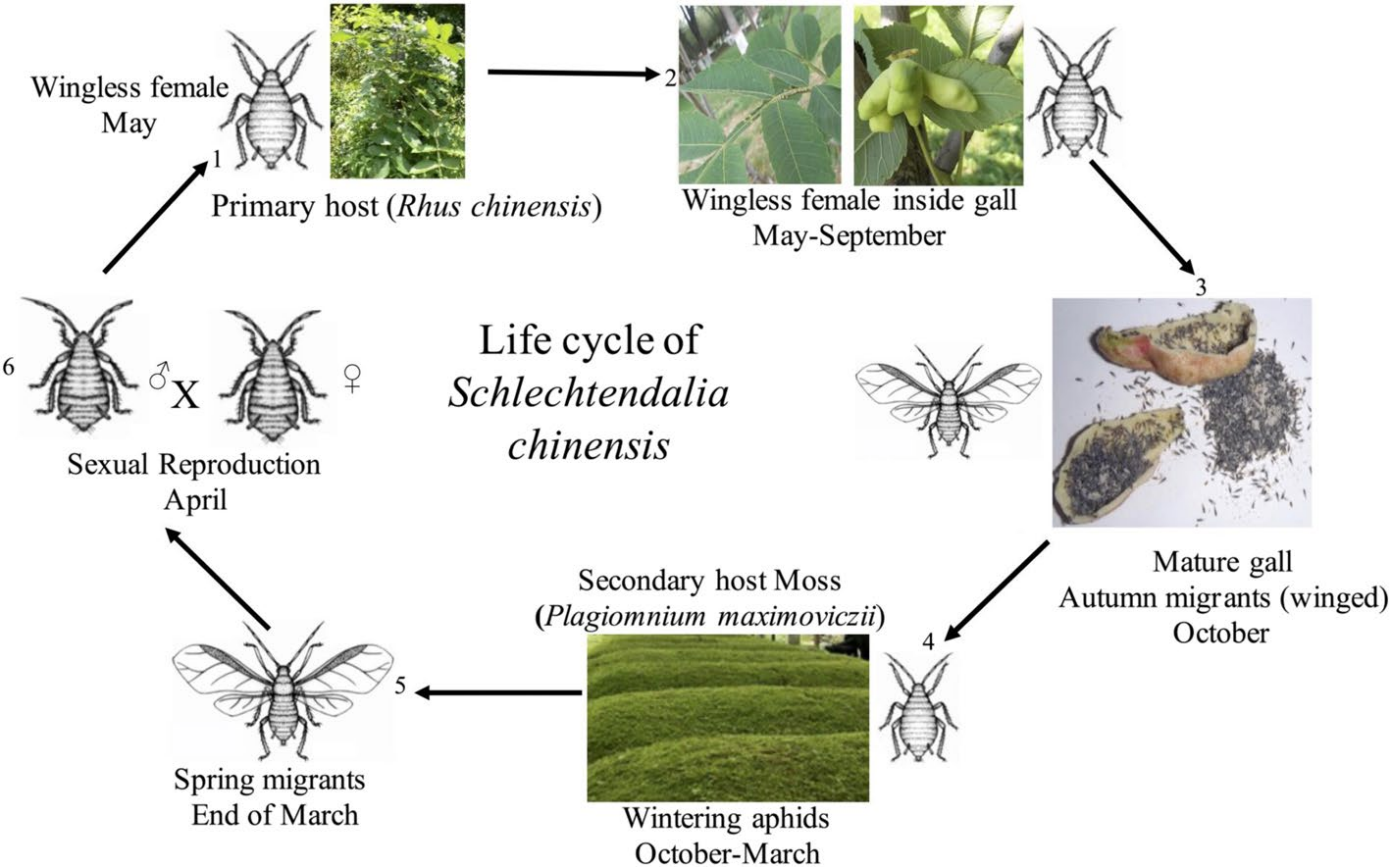
The life cycle of *Schlechtendalia chinensis* comprises several generations and alternates between two unrelated host plants. (1) A wingless foundress is produced on its primary host *Rhus chinensis*. (2) The foundress induces gall on leaves, feeds and lives inside the gall, and reproduces wingless daughters which also reproduce for several generations parthenogenetically from April-September. (3) Winged autumn migrants emerge from the mature gall and fly to nearby moss *Plagiomnium maximoviczii*. (4) The migrants reproduce in moss, and their offspring feed and live in moss from October-March, (5) Winged spring migrants fly to *R. chinensis* and reproduce sexual males and females. (6) Sexual offspring mate in the trunk cervices and reproduce female foundresses, which crawl to the leaves and start the next life cycle

*Schlechtendalia chinensis* is one of the widely distributed species of *Rhus* gall aphids in China and induces horned-shaped galls (“gallnuts”) on the leaves and shoots of its primary host plant, *Rhus chinensis* (Anacardiaceae). This aphid species undergoes host alternation between *R. chinensis* and mosses (*Plagiomnium spp*. Mniaceae). In this life cycle, a foundress produced by a mated female on a *Rhus* tree settles on a developing leaf and begins to feed. Through effectors introduced by her saliva, she induces the formation of a horn-gall, which serves as a sheltered micro-environment for feeding and reproduction [18]. The aphids reproduce parthenogenetically for three generations in the gall, which grows to form a highly organized structure. Galls mature and form openings in autumn, releasing adult autumn migrants that fly to nearby moss, where they give birth to nymphs that live there overwinter. Nymphs develop into adult spring migrants in the following spring and fly back to *R. chinensis*, producing sexual male and female offspring. After mating, each female produces a foundress to begin the life cycle again (Fig. 1) [1, 18, 19]. As in other host-alternating aphids, *S. chinensis* produces a series of all-female parthenogenetic generations with only a single sexual generation.

The galls induced by *Schlechtendalia chinensis* are rich in tannin, which ranges from 50% to 70% in dry weight [20]. The galls have economic importance and have been used for different applications in the medical, food, and chemical industries, and for military purposes [7, 13]. Thus, gall farming has become one of the growing agricultural industries in China, and the annual yield of gallnuts is eight to ten thousand tons, accounting for >90% of the total yield worldwide [13, 18]. Because the global human population is estimated to reach up to nine billion by 2050, the demand for food and other commercial products is expected to increase by more than 70% [21, 22]. Galls produced by *S. chinensis* can be considered a sustainable alternative for many products.

Although gall-forming aphids have life cycles and associated adaptations that are distinctly different from other aphids and have practical economic importance, there is still a considerable knowledge gap concerning this category of aphids, particularly at the genomic level. There is very little published whole genome information available for the gall-forming aphid species among the 20 published genomes of aphids. Only recently, a genome of gall aphid *S. chinensis* was published [9], when this project was in progress, but didn’t cover all the key aspects of the genome that we examined in this study.

In this study, we generated a high-quality chromosome-level genome assembly of the *Rhus* gall aphid, *S. chinensis* (“horn-gall” aphid), which represents a completely and deeply sequenced genome of a gall-inducing aphid. We used a combination of Illumina, PacBio, and High-throughput Chromosome Conformation Capture (Hi-C) technologies. We carried out gene prediction and mining, functional annotation, comparative and phylogenetic analysis of the sequenced genome of *S. chinensis* with other aphid (Aphididae) species. The genome of *S. chinensis* will serve as a resource for detailed information about the genomic organization of gall aphids in the Eriosomatinae. It will also allow us to better identify genes associated with diverse developmental processes, including gall induction on the primary host plant and interactions with the secondary host plant. This study also provides insights into the endogenization of *parvovirus*-related genes and a detailed analysis of the P450 family in the *S. chinensis* genome, which could have an essential role in the evolution and adaptation of these aphids.

## Materials and methods

### Sample collection and DNA extraction

We collected the fresh, mature gall formed by *S. chinensis* on *Rhus chinensis* in Wufeng county (30°19′ N, 110°67′ E, 329 m above sea level), Hubei, China, in October, 2019. For genomic DNA (gDNA) extraction, about 200 individual winged females derived in one gall by parthenogenesis from a single clone were obtained by dissecting the gall and separating aphids from impurities in a petri dish. Insects were subsequently frozen in liquid nitrogen, and whole-genome DNA was extracted using a DNeasy extraction kit (QIAGEN, Valencia, CA). Another about 100 aphids from the same gall were obtained to extract the gDNA for Hi-C sequencing repeating the same protocol. The quality of the DNA was assessed by gel electrophoresis. The plant leaves and galls used in this study was formally identified by the corresponding author and the specimens were stored at the laboratory facility and didn’t deposit to any public herbarium.

### Illumina and PacBio library construction and sequencing

Genomic DNA was used to construct DNA libraries for draft assembly. Two libraries of 350 bp were constructed through physical fragmentation by ultrasonic shock for Illumina 3^rd^ generation sequencing on the Illumina HiSeq2500 platform. Using 350 bp DNA libraries provides sufficient distance between the paired reads and facilitates better genome assembly and mapping. We also selected a 350 bp insert size for libraries because of its compatibility with the HiSeq2500 platform for better genome assembly. Quality inspection of libraries was done using Q-PCR to detect fragment size and quantity. Both libraries were sequenced using paired-end 2x150 bp reads to generate a total of 85 Gb with a raw data depth of 206.21x, i.e., 36 Gb (86x) and 49 Gb (120x). After quality control, 35.31 Gb of data were kept for draft assembly improvement. Estimation of genome size, repeat sequence ratio and heterozygosity was performed using the 350 bp library data to build a k-mer distribution map with k=19.

For long-read sequencing, high-quality genomic DNA extracted from *S*. *chinensis* was used to prepare genomic libraries following the standard protocol provided by PacBio, including sample quality testing, library building, quality inspection, and sequencing. DNA was sequenced on the PacBio Sequel II sequencing platform. Highly accurate long reads (HiFi) were produced using the circular consensus (CSS) mode, which resulted in the generation of long reads (raw data), which were then used to produce clean data.

### Hi-C sequencing and scaffolds assembly

We constructed Hi-C fragment libraries from 300-700 bp insert size as Rao et al. illustrated and sequenced on the Illumina platform [23]. Briefly, adapter sequences of raw reads were trimmed, and 150 bp pair ends of low-quality reads were removed to obtain clean data. The clean Hi-C was first truncated at the putative Hi-C junctions, and then the resulting trimmed reads were aligned to the assembly results with the BWA aligner [24]. Only uniquely aligned pairs of 140,590,231 (77.7%) reads with the best mapping quality were retained for further analysis. Invalid read pairs, including dangling-end and self-cycle, re-ligation, and dumped products, were filtered by HiC-Prov2.8.1 [25].

The 83,713,688 (59.54%) unique mapped read pairs were valid interaction pairs used to correct scaffolds and cluster, order, and orient scaffolds onto chromosomes by LACHESIS [26]. Before chromosome assembly, we first performed a pre-assembly for error correction of scaffolds, which required splitting scaffolds into segments of 50 kb on average. The Hi-C data were mapped to these segments using BWA v0.7.10-r789 software [24]. The uniquely mapped data were retained to perform assembly using LACHESIS software [26]. Any two segments that showed an inconsistent connection with information from the raw scaffolds were checked manually. These corrected scaffolds were then assembled into 13 chromosomes with LACHESIS. Position and orientation errors exhibiting obvious discrete chromatin interaction patterns were manually adjusted. In the end, 29 scaffolds (representing 91.7% of total length) were anchored into 13 chromosomes.

Hi-C sequencing is a powerful technique used in genomics that enhances chromosome-level genome assembly by providing information about the spatial organization and interactions of DNA sequences within the three-dimensional structure of the nucleus. Hi-C sequencing assists in scaffolding or linking together contigs from short-read sequencing methods. This helps in ordering and orienting these sequences along chromosomes, thereby improving the continuity and accuracy of chromosome-level genome assemblies [23]. Hi-C data was used to create chromosome conformation maps that illustrate the spatial relationships between genomic loci. These maps provide a visual representation of chromosomal interactions, aiding in the interpretation of genome assembly and structure.

### Gene prediction and annotation

We integrated three approaches, namely, *de novo* prediction, homology search, and transcript-based assembly, to annotate protein-coding genes in the genome of *S. chinensis*. The *de novo* gene models were predicted using two ab initio gene-prediction software tools, Augustus v2.4 and SNAP (2006-07-28) [27, 28]. For the homolog-based approach, GeMoMa v1.7 software was run using a reference gene model from another species [29]. Full-length transcripts from the PacBio sequencing were assembled using Trinity v2.11 [30, 31]. Gene models from these approaches were combined using the EVM software v1.1.1 and updated by PASA [32]. The final gene models were annotated by searching the GenBank non-redundant database nr, TrEMBL, Pfam (33.1), SwissProt, eukaryotic orthologous groups (KOG), gene ontology (GO) [33], and Kyoto Encyclopedia of Genes and Genomes (KEGG) [34] databases. We performed gene prediction using the default parameters and thresholds for each of the pipelines and tools used in the analysis.

### Transposable elements and tandem repeats

Transposable elements (TEs) and tandem repeats were identified and annotated by combining homology-based and de novo approaches. We first customized a genome de novo repeat library using RepeatModeler [35], which can automatically execute two de novo repeat finding programs, including RECON v1.08 [36] and RepeatScout [37]. Full-length long terminal repeat retro-transposons (LTR-RTs) were identified using both LTRharvest with parameters (minlen: 100, maxlen: 40000, mintsd: 4, maxtsd: 6, motif: TGCA, motifmis 1, similar 85, vic 10 -seed 20, seqids yes) and LTR_finder with parameters (D 40000, d 100, L 9000, l 50, p 20, C -M 0.9) [38, 39]. The high-quality intact LTR-RTs and non-redundant LTR library were then produced by LTR_retriever [40]. A non-redundant species-specific TE library was constructed by combining the de novo TE sequence library with the known Repbase v19.06, REXdb v3.0, and Dfam v3.2 databases [41–43]. Finally, TE sequences in the *S. chinensis* genome were identified and classified by homology search against the library using RepeatMasker v4.10 [44]. Tandem repeats were annotated by Tandem Repeats Finder [45] and the Microsatellites identification tool MISA v2.1 [46].

### Pseudogene prediction and non-coding RNAs

Pseudogenes usually have similar sequences to functional genes, but may have lost their biological function because of genetic mutations causing frameshifts or premature stop codons. The GenBlastA v1.0.4 program was used to scan the whole genome after masking predicted functional genes [47]. Putative candidates were then analyzed by searching for nonsense mutations using GeneWise v2.4.1 [48].

Non-coding RNAs are usually divided into several groups, including miRNA, rRNA, tRNA, snoRNA, and snRNA. The tRNAscan-SE v1.3.1 was used to predict tRNAs with eukaryote parameters [49]. Identification of the rRNA genes was conducted by barrnap v0.9, and miRNA were identified by searching miRBase (released 21) databases, while snoRNA and snRNA genes were predicted using Infenal v1.1 against the Rfam (released 12.0) database [50–53].

### Phylogenetic analysis

A phylogenetic tree of *S. chinensis* with ten other aphids and the whitefly, *Bemisia tabaci* (Aleyrodoidea), as the outgroup was reconstructed from 1901 single-copy orthologous genes using IQ-TREE v1.6.11 software [54]. Sequences of all species were aligned using MAFFT v7.205 [55], followed by Gblocks v0.91b (parameter: b5-h) [56]. jModelTest was used for model detection, and the best-fit model JTT+ F+I+G4 was used to estimate the phylogenetic tree under maximum likelihood (ML) with 1000 bootstraps [57]. PAML v4.9i software [58] was used to calculate the divergence time. Specifically, we used the TimeTree (http://www.timetree.org/) website to estimate the divergence times between the studied species. After the calibration of specific nodes, divergence times were estimated with the MCMCTREE [59] module in PAML using the correlated molecular clock and JC69 models. The resulting chronogram was graphically displayed using MCMCTreeR v1.1 [59]. Phylogenetic analysis of PRDs and the P450 gene family was performed using the above steps, and the tree was constructed using IQ-TREE software, with best-fit model GTR+I+G.

### Gene family expansion/contraction and positive selection

We used cafes v4.2 [60] to estimate the number of gene family members for each hypothetical ancestor using the chronogram and gene family clustering. Thus, the contraction and expansion of the gene family relative to the species' ancestors are predicted. The criteria we defined for significant expansion or contraction were family-wide p values and Viterbi p values to be less than 0.05. Rapidly evolving genes show expected non-synonymous mutations (K_a_) greater than synonymous mutations (K_s_), i.e., K_a_/ K_s_ value greater than 1. We primarily used the codeML modules in PAML [58] for positive selection analysis of the single-copy gene families of *S. flava*, *S. chinensis*, *R. maidis*, *A. pisum*, and *M. persicae*. Then, we used MAFFT [55] to compare the protein sequences of each gene family, followed by PAL2NAL inversion to cipher pairing sequences [61]. Finally, CodeML (using F3x4 model of codon) frequencies based on the Punch-site model, through the "chi2" program under PAML, were used to detect significant differences (*p*-value <0.05) [58]. Based on the branch-site model, we chose the two models Model A (assuming that the foreground branches are in positive selection, i.e., s >1) and null Model (LRTs, likewise test <s) based on the "chi2" program under PAML [58]. We used the Bayesian method ( BEB, Bayes empirical Bayes method) [62] to obtain the post-test probability (usually greater than 0.95 is considered to indicate significantly positively selected sites) to compile a list of genes that were significantly positively selected.

### Collinear analysis

The collinear analysis of genes can unveil the signature of genome structure variation. In addition, the gene pairs of homologous origin can be obtained through collinear analysis. We performed the collinear analysis using DIAMOND v0.9.29.130 [63] to determine similar gene pairs (e<1e-5, C score >0.5). Distance between identical gene pairs on chromosomes was determined primarily through MCScanX (parameter-m 15) [64], resulting in all the genes in the collinear block. The collinear analysis of *S. chinensis* was performed with *A*. *pisum* as reference genome, and used JCVI v 0.9.13 [65] to draw the collinear figure.

### Whole genome duplication

Whole Genome Replication Event (Whole Genome duplication, WGD), also known as ancient multiplication, is the process of doubling the overall content of the genome. The whole genome doubling event produces many paralogous genes, resulting in significant K_s_ value and high K_s_ value peaks corresponding to the whole genome doubling events. We used the commonly used methods to identify WGD, i.e., K_s_ method and 4DTv method., and determined WGD in *S. chinensis* by mapping K_s_ and 4DTv curves within and between the species. We used software wgd v1.1.1 [66] to calculate synonymous mutation rate (K_s_). We used the Script (https://github.com/JinfengChen/Scripts) to calculate the proportion of each paralogous gene that had a mutation in the base of the 4DTv site and to correct it using the HKY alternative model to obtain the results and plot.

## Results

### Chromosome-length scaffold assembly of *Schlechtendalia chinensis*

Our strategy of genome assembly employed Illumina (San Diego, CA) short read and PacBio (Menlo Park, CA) long-read sequencing data with scaffolding informed by high-throughput chromosome conformation capture (Hi-C) [67]. Aphid samples were collected from the gall induced by *S. chinensis* on *Rhus chinensis* and DNA was extracted for sequencing from 200 autumn migrant individuals. We constructed two DNA libraries of 350 bp, followed by sequencing on the Illumina HiSeq 2000 paired-end technology resulting in the generation of 85 Gb and 236,191,478 raw reads. Finally, 35 Gb of clean reads were produced after removing low-quality reads, corresponding to ~86-fold coverage of the haploid genome. The genome size was estimated to be 409.55 Mb by K-mer analysis (k=19) and estimated heterozygosity of 1.31%, which indicated a highly heterogeneous complex genome (Additional file 1: Figure S1). In addition, the sequencing from PacBio libraries on the PacBio PS II platform yielded 510 Gb of raw data. After quality checking and removing the reads of endosymbionts, a total of 36.04 Gb clean data was produced corresponding to ~90-fold coverage of the genome from 2,257,809 reads with N50 of 16,203 bp, which indicated the high quality of this sequenced genome (Additional file 1: Table S1 and S2).

**Fig. 2:**
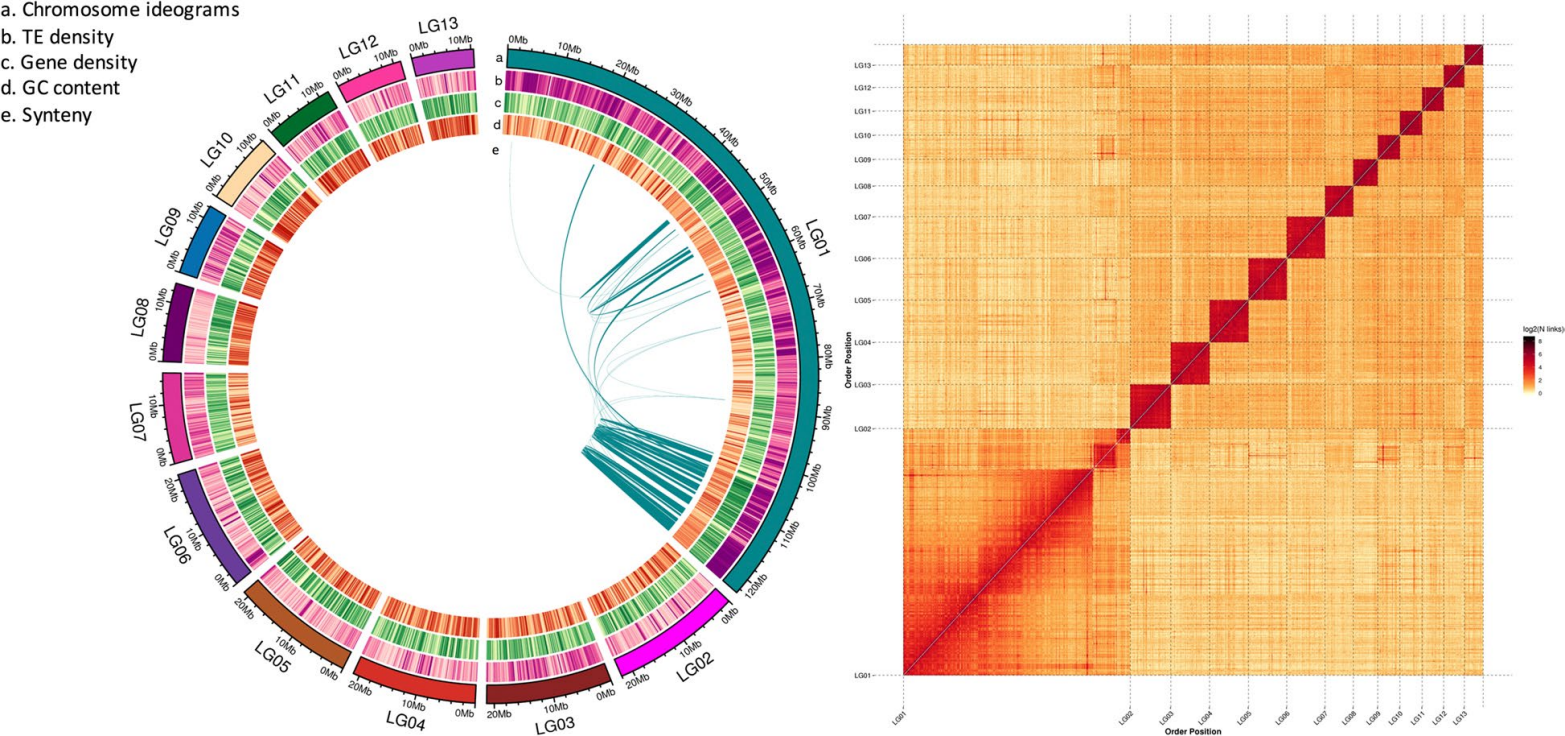
Chromosome-level genome assembly of *Schlechtendalia chinensis*. **A** Genomic landscape of the 13 *S. chinensis* chromosomes (LG1–LG13 on a Mb scale). Each track notation is listed in the center of the circle. **B** Heatmap showing the frequency of Hi-C contacts along with the *S. chinensis* genome assembly. Genome scaffolds are ordered from longest to shortest with the x- and y-axis showing order position

Hi-C generated 53 Gb of data consisting of 180,735,576 reads, combined with the PacBio assembly, further improved data quality for assembling 189 scaffolds with N50 (210,091,861 bp). All 189 scaffolds were anchored and oriented onto 13 chromosomes, in which more than 91.71% of assembled sequences were located (Fig. 2, Table 1, Additional file 1: Table S3). We did not encounter chromosomal super- scaffold gaps after Hi-C scaffolding, indicating the high genome assembly quality (Additional file 1: Table S4). To evaluate the completeness of the genome assembly, BUSCO analysis was performed against arthropod datasets of 939 metazoan species, and 97% of *S. chinensis* genes were mapped to the reference genomes, which validated the completeness and high quality of our genome (Table 1, Additional file 1: Table S5). Our assembly of the *S. chinensis* genome has almost the same size as those of other aphid genomes publicly available.

**Table 1:** Detailed statistics of the *Schlechtendalia chinensis* genome assembly, showing the assembly features and gene models

**Assembly features**	**Hi-C Scaffolds**
Number of chromosomes (n)	13
Number of Scaffolds	189
Total size of Scaffolds	344.56 Mb
Longest scaffold size (Mbp)	122.78 Mb
Shortest Scaffold size (Mbp)	15.65 Kb
Mean scaffold length (Mbp)	1.82 Mb
Median scaffold size	77.06 Kb
N50 scaffold length	21.09 Mb
L50 scaffold count	10
Scaffolds GC content	33.74%
Scaffolds Gaps (N) content	0
Percentage of assembled contigs in scaffolds	96.92%
Average number of contigs per scaffold	1.03
BUSCO (complete)	94.34%
**Gene models**	
Number of genes models	15,289
Mean coding sequence length CDS	1520.26 bp
Mean number of exons per gene	6.61
Mean exon length	1822.17 bp
Mean intron length	6663.72 bp
Non-protein-coding genes	426
Number of miRNA gene	23
Number of tRNA gene	136
Number of rRNA gene	33
Number of snRNA gene	71
Number of soRNA	13
**Pseudogenes**	
Number of pseudogenes	192
Total length	523,288
Average length	2725.46

### Gene prediction and genome annotation

In total, 129,303,997 bp repeat sequences were identified, spanned ~130 Mb, and constituted 37.52% of the *S. chinensis* genome (Additional file 1: Table S6). We predicted 15,013 genes in the genome using the program EVidenceModeler (EVM), which used the combination of Ab initio gene prediction, protein, and transcripts alignment into predicted gene structure (Additional file 1: Figure S2, Table S7). The number of predicted genes in the *S. chinensis* genome is within the range of other aphid genomes (11,980-32,005) but is on the lower end (Table 2). However, because the number of predicted genes might vary depending on the quality of the genome and prediction pipeline [68, 69], we further used a collection of eight annotation databases, i.e., KEGG, KOG, Pfam, GO, TrEMBL, eggNOG, Nr, and Swissprot, to annotate protein-coding genes. In the end, we obtained annotation for 14,582 (97.13%) coding genes, 99% of which were anchored to the 13 chromosomes. The average sequence lengths for the entire gene regions, exons, coding regions (CDS), and introns are shown in Table 1.

**Table 2:** Comparative whole genome phylogenomic analysis of *Schlechtendalia chinensis* with the genomes of 10 aphid’s species included in the study

	*S*. *chinenisis*	*A*. *craccivora*	*A*. *glycine*	*A*. *gossypi*	*A*. *pisum*	*C*. *cedri*	*D*. *noxia*	*M*. *persicae*	*M*. *sacchari*	*R*. *madis*	*S*. *flava*
No. of genes	15,013	32,005	18,358	12,343	18,214	16,684	12,290	14,825	12,269	11980	13,504
Genes in orthogroup	13,596	23,856	13,092	11,496	15,815	13,021	11,157	14,001	11,795	11,530	12,364
Percentage of genes in orthogroup	90.6	74.5	71.3	93.1	86.8	76.7	90.8	98.4	96.1	96.2	91.6
Unassigned genes	1,417	8,149	5,266	847	2,399	3,963	1,133	824	474	450	1,140
Percentage of unassigned genes	8.4	25.5	28.3	6.9	13.2	23.3	8.2	1.6	3.9	3.8	8.4
Number of orthogroups containing genes	9,865	13,049	9,561	9,909	11,324	10,080	9,788	10,811	10,161	9,844	9.817
Species-specific orthogroup	193	832	221	15	211	361	14	56	28	44	103
Genes in Species-specific orthogroup	893	4,105	993	31	601	1,069	31	138	80	123	354
Percentage of genes in Species-specific orthogroup	2.6	12.8	5.4	0.3	3.3	6.3	0.3	0.9	0.7	1.0	5.9

The number of the predicted protein-coding genes in the *S*. *chinensis* genome (15,013) is closest in range to the number of genes annotated for *Cinara cedri* (16,684) and *Myzus persicae* (14,825), but less than half that of *A. craccivora* (32,005) (Table 2). Among the 15,013 predicted genes, 14,582 (97.13%) could be annotated by one of the eight protein-coding databases, i.e., GO 10,037 (66.86%), KEGG 10,997 (73.25%), KOG 7,762 (51.7%), Pfam 11,447 (76.25%), TrEMBL 14,546 (96.89%), eggNOG 10,795 (71.9%), Swissprot 9,847 (65.59%) and the NCBI non-redundant protein database nr 14,238 (97.13%) (Additional file 1: Table S8).

We predicted the functional classification of the annotated genes using the eggNOG database, which returned 3,845 (33.5%) of the genes as having unknown functions (Additional file 1: Figure S3). Subsequently, we predicted different types of non-coding RNAs (ncRNAs), including small nuclear RNAs, tRNAs, rRNAs, micro RNAs (miRNA), and small nucleolar RNAs (snoRNAs), from a small RNA library (Table 1). Furthermore, a total of 192 pseudogenes were identified in the assembled genome of *S. chinensis* (Table 1).

### Comparative genomics and phylogeny

**Fig. 3:**
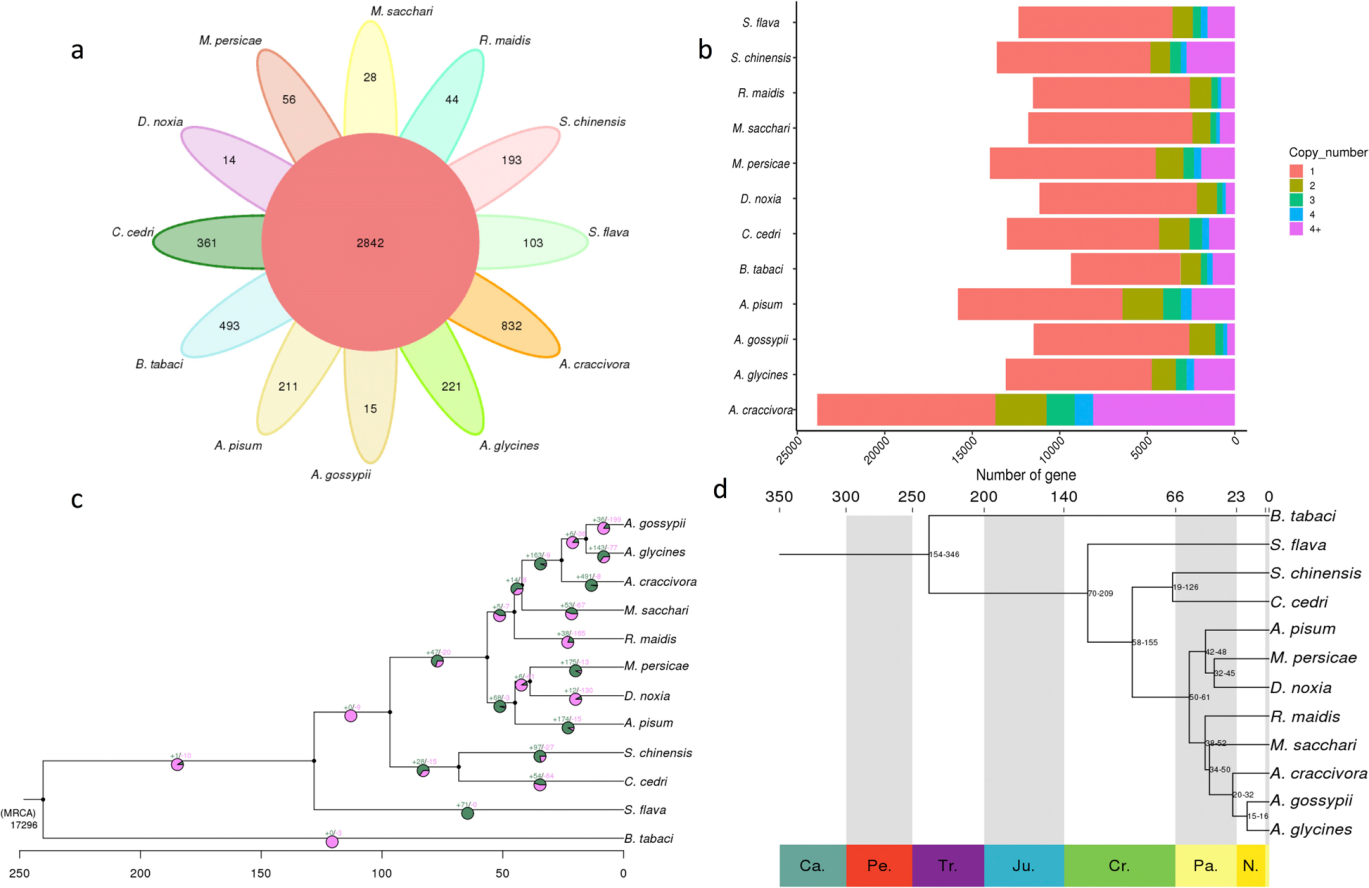
Comparative genomics and evolutionary analysis of *Schlechtendalia chinensis*. **A** Petal diagram shows common and shared gene families of *S. chinensis*. The middle circle is the number of gene families common to all species, and the petals show the number of gene families unique to each species. **B** The number of gene copies in each gene family of each species, including the number and proportion of gene families containing from zero to four copies and more than four copies. The X-axis shows the number of the gene family, while the Y-axis shows each species and copy number of genes. **C** A Pie charts at each node indicate the proportion of gene families contracted and expanded in each branch of the evolutionary tree. Note: "+" represents the number of gene families expanded, and "-" represents the number of gene families contracted. **D** Phylogenetic tree constructed using 1902 single-copy gene sequences, with IQ-TREE v1.6.11. Numbers at the nodes are divergence times supported by 95% hpd (highest posterior density). Below the tree is the geological timescale; above the tree is the absolute age, in millions of years, and the shaded areas define each geological period

We downloaded whole genomes of 10 species of aphids and one whitefly species (*Bemisia tabaci*) as an outgroup for comparative genomic analysis (Additional file 1: Table S9). We clustered the annotated genes to identify gene families (orthogroups) and genes common to all species. We analysed 19,874 orthogroups and found that a total of 13,596 (90.6%) genes in *S. chinensis* clustered into 9,865 orthogroups, among which 893 genes (5.9%) belonged to 193 *S. chinensis*-specific orthogroups (Table 2). The number of orthogroups unique to *S. chinensis* vs. shared by all 12 species and the copy number of genes for each species was also analysed (Fig. 3A and B). The genes specific to *S. chinensis* were also analysed by GO and KEGG enrichment analyses for their functions using cluster Profiler v3.14.0 [70]. They were characterized as involved in molecular functions, various biological processes, and related to structural cellular components (Additional file 1: Figure S4).

The prediction of contraction and expansion of the gene families relative to the other 11 species showed a higher frequency of gene expansion in the *S. chinensis* genome. Compared with other aphid species in the analysis, 97 gene families in *S. chinensis* underwent expansion, while only 27 gene families underwent contraction (Fig. 3C). Using GO pathway enrichment analysis, the 97 expanded gene families were found to have a role in DNA repair, telomerase maintenance, DNA helicase activity, protein dimerization, RNA-directed polymerase activity, and aspartic-type endopeptidase activity (Additional file 1: Figure S5). In contrast, the roles of 27 contracted gene families were related to the oxidation-reduction process, nucleosomes assembly, and methylation (Additional file 1: Figure S6).

**Fig. 4:**
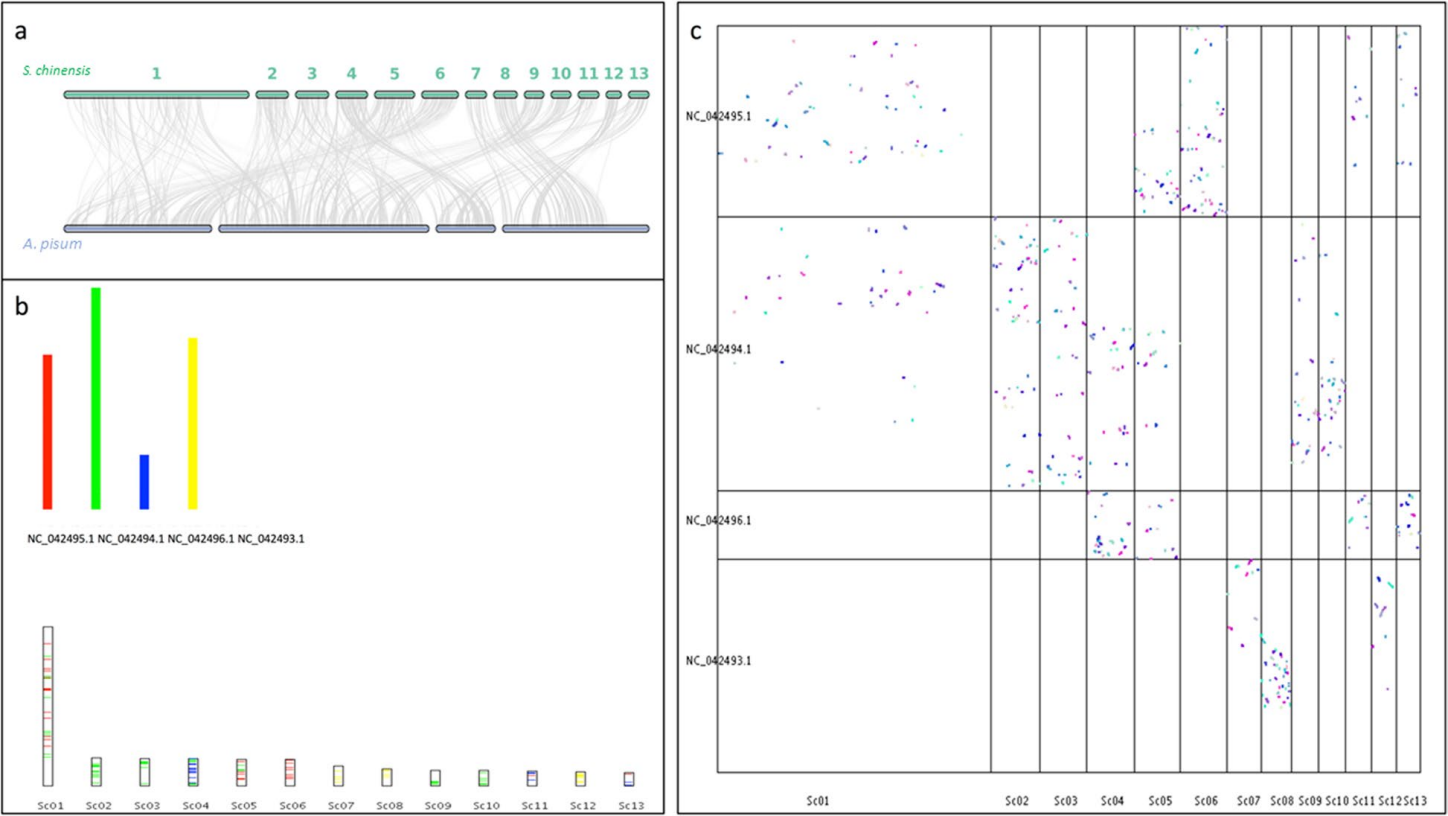
Comparative collinear analysis of *Schlechtendalia chinensis* with *A. pisum*. **A** Collinear analysis and synteny between *S. chinensis* and *A. pisum*. This figure shows that the number of non-primary corresponding chromosomal collinear genes is fewer than 100 (intraspecific collinearity is not filtered). **B** Collinear analysis of the individual chromosomes in *A. pisum* and *S. chinensis*. **C** Syntenic blocks between *A. pisum* and *S. chinensis*. The X-axis represents *S. chinensis* and the Y-axis represents A. *pisum* chromosomes

Collinear analysis of the *S*. *chinensis* genome with a reference genome (*Acyrthosiphon pisum*; GenBank accession (PRJNA547584) was performed to evaluate genome structure variation by using DIAMOND v0.9.29.130 [63], and to determine similar gene pairs. Collinear analysis was performed to analyze variation between *S. chinensis* and the reference genome from Aphidoidea, which could help to verify the accuracy of genome assembly. In addition, collinearity analysis is helpful to obtain gene pairs of homologous origin, which simplifies the calculation of K_a_/ K_s_ for genome duplication analysis, as collinear genes tend to have the same biological function. Based on all predicted and annotated genes in the *S. chinensis* genome, collinear genes between chromosomes were determined using MCScanX [64] (parameter-m 15). All the genes were arranged in the collinear block, and the linear pattern of all genes of *S. chinensis* against the reference genome was predicted (Fig. 4A and B). The collinearity analysis compares all the orthologs shared by *S. chinensis* with the reference genome, with random distribution on chromosomes because of different sizes and numbers of genomes and chromosomes, respectively.

We reconstructed a Maximum Likelihood (ML) phylogenetic tree by selecting 1,902 single-copy orthologous genes from the 11 aphid species with *B. tabaci* as an outgroup to root the tree (Fig. 3D) using IQ-TREE v1.6.11 software [54]. We calculated the aphid divergence time using TimeTree [71] (http://www.timetree.org/) (see Materials and methods for details). The phylogenetic analysis and estimated divergence time indicate that, among this limited sample of aphid diversity, *S*. *chinensis* is most closely related to *C. cedri* and diverged from the latter around 19-126 million years ago (MYA); Eriosomatinae+Lachninae diverged from Aphididae around 58-155 MYA (Fig. 3D).

### Whole genome duplication and positive selection

**Fig. 5:**
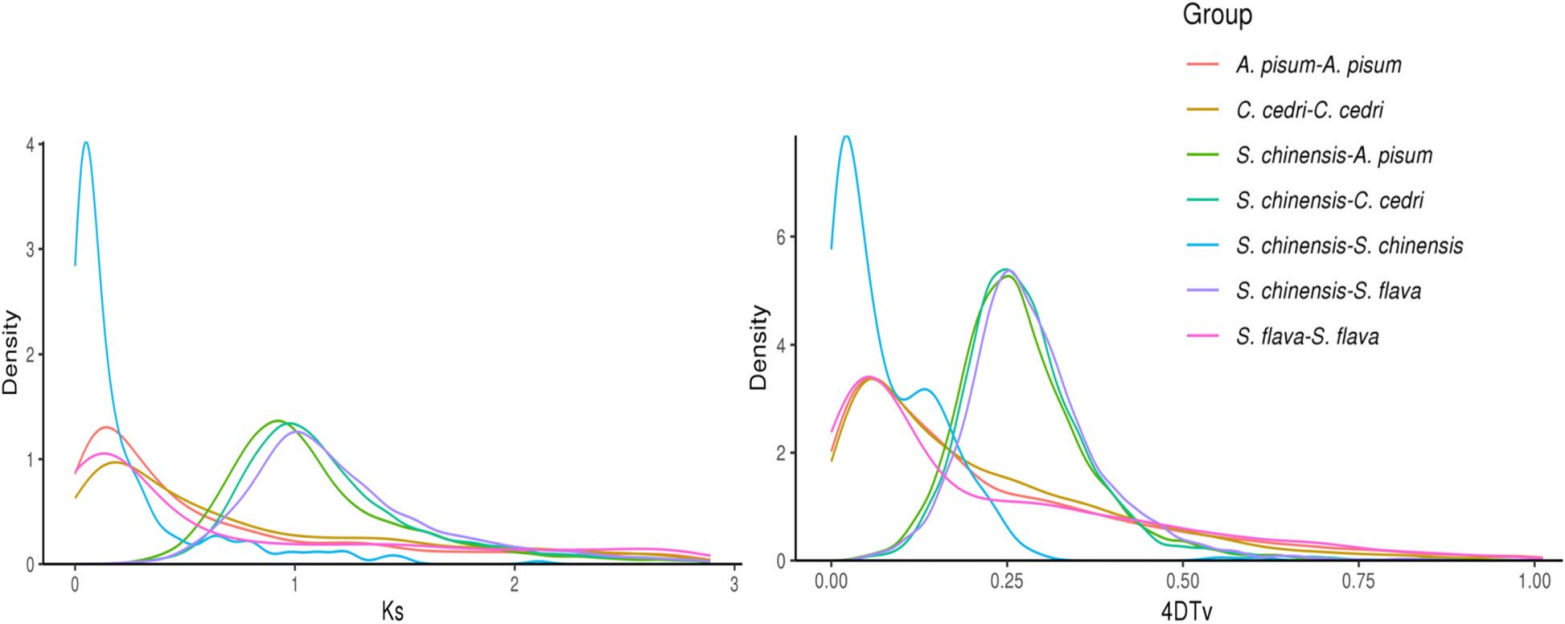
Whole-genome duplication analysis of *S. chinensis*. Genome duplication events were revealed by the high K_s_ and 4DTV distribution peaks

Whole Genome Duplication (WGD) is the process of doubling the overall content of the genome and has a significant impact in shaping the evolution of species. Ancient WGDs have been associated with major eukaryote lineages, and many events of WGD have been detected in insects [72]. To evaluate the possibility of WGD in *S. chinensis*, we analyzed the distribution of synonymous substitution rates per gene (K_s_) and four-fold synonymous (degenerative) third codon transversion (4DTv) between collinear paralogous genes. We mapped the K_s_ and 4DTv curves within and between the species (*S. chinensis*, *A. pisum*, *C. cedri*, and *S. flava*) to determine WGD occurrence. One prominent peak was observed in the *S. chinensis* genome based on the abundance of K_s_ sites values (K_s_ value of 0.25) and 4DTv value (4DTv value of 0.05), indicating that *S. chinensis* had experienced a WGD event during its evolution. The genomes of *A. pisum*, *C. cedri,* and *S. flava* were used to identify the K_s_ and 4DTv values collinear blocks between *S. chinensis*, which suggested that *S. chinensis* experienced large-scale duplication more recently than these three aphid species (Fig. 5).

Based on K_a_/K_s_ value, we performed an analysis for positive selection and identified nine genes from different gene families containing significantly positively selected sites (Additional file 1: Table S10). When genes are strongly positively selected, they may result in new functions for species [73]. Positively selected genes are the foundation for new functions in species and have a prominent role in evolution. GO enrichment analysis revealed that positively selected genes in *S. chinensis* were related to the "transcription regulation" category of biological processes, "transport vesicles and plasma membrane" in the cellular component category, and "sequence-specific DNA binding and transcription regulator activity" in the molecular function category (Additional file 1: Figure S7). While the KEGG pathway enrichment analysis showed that these positively selected genes were mainly related to neuroactive ligand-related reception, apoptosis, and the phosphatidylinositol signalling system (Additional file 1: Figure S8).

### Endogenization of *Parvovirus* like DNA sequences

**Fig. 6:**
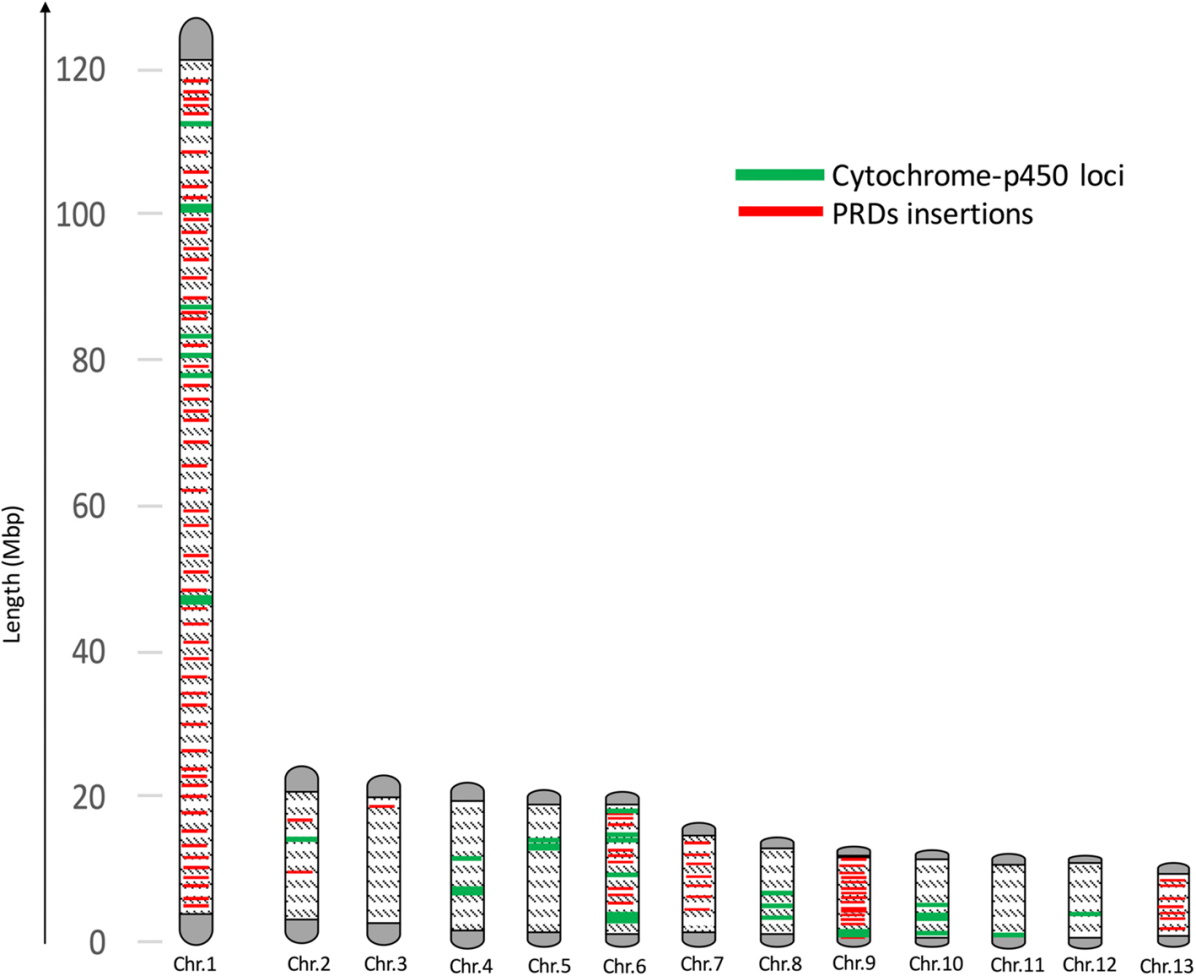
Localization of *Parvovirus*-like DNA sequences (PRDs) and cytochrome P450 protein genes on 13 chromosomes of *S. chinensis*. Red lines represent PRDs insertions and green lines represent cytochrome P450 genes

*Parvoviruses* (*Parvoviridae*) are single-stranded DNA viruses that infect a wide variety of arthropods and insects, including aphids [74]. *Densoviruses* (*Parvoviridae*: *Densovirinae*) have been reported to infect aphid species, e.g., *Myzus persicae* and *Dysaphis plantaginea* [75, 76]. Integration of *Densovirus* genes, including structural and non-structural types, has also been reported in non-galling aphid species [74, 77]. Using EVM gene prediction followed by multiple annotation software (see Materials and methods), we found many DNA sequences related to *Parvoviruses* in the genome of *S. chinensis*. We analyzed all the predicted *Parvovirus*-like DNA sequences (PRDs) against the Pfam database, which classified all the sequences into their respective groups and families. A significant number of PRDs were classified as "*Parvovirus* coat protein VP1" genes. We identified a total of 115 PRDs integrated into 13 chromosomes of the *S. chinensis* genome (Fig. 6), while few PRDs were found in unanchored scaffolds. All the analyzed PRDs have variable lengths (519-3204 bp) with 2-3 exons and 1-2 introns in each sequence.

We performed functional annotation of the PRD peptide-coding sequences (CDS) using GO functional annotation and KEGG functional annotation databases. GO annotation showed that some of the PRDs have phospholipase A2 activity (GO:0004623) in the molecular function category, and phospholipid metabolic process (GO:0006644) and arachidonic acid secretion (GO:0050482) in the category of biological process (Additional file 2). At the same time, KEGG annotation showed that these sequences have a role in amino acid metabolism, i.e., lysine degradation and histone (H3)-lysine N-methyl transferase SETMAR activity (K11433). The functional activity of these sequences might also reflect stable domestication of these sequences in the host genome, like SETMAR protein which is the product of domesticated transposase fused with methylase [78].

Based on the above analysis, further investigations will be required to find the specific function of these sequences. Other databases, i.e., TrEMBL and nr, annotated the PRDs in *S. chinensis* as uncharacterized and hypothetical proteins in *Aphis glycines*, *Acyrthosiphon pisum,* and a protein of *Tribolium castaneum* (Additional file 2). The presence of PRDs in other aphids reflects the old and stable integration of these sequences in aphids, possibly by horizontal gene transfer. We did not find any non-structural genes related to *Parvoviruses* in the genome of *S. chinensis*.

**Fig. 7:**
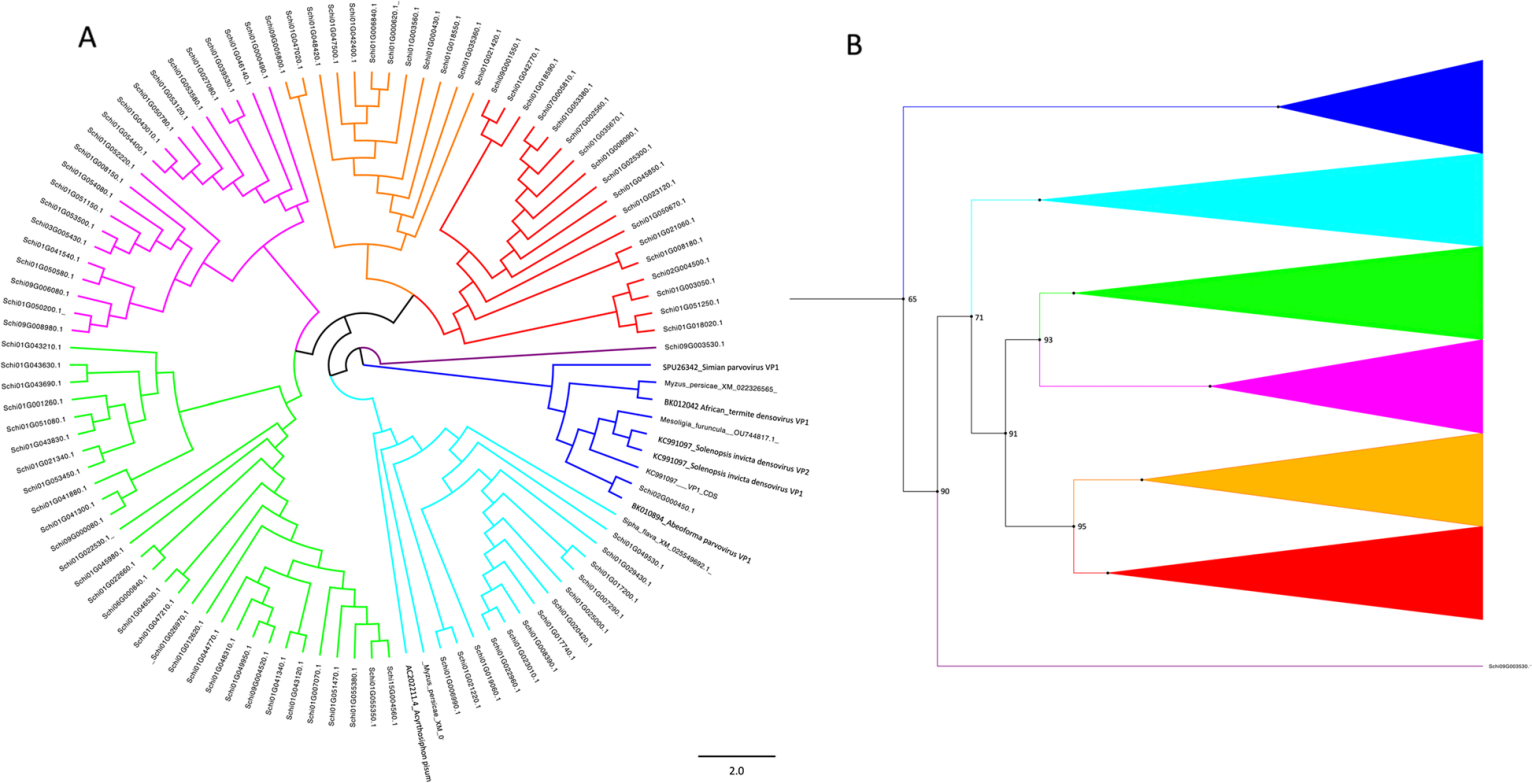
Phylogenetic relationships of endogenized PRDs in the *Schlechtendalia chinensis* genome. **A** Figure showing the phylogenetic relations of all sequences, and color represents a different clade of PRDs, indicating independent evolutionary origins. Tips of the branches represent the gene I.D.s for *S. chinensis* sequences and GenBank accession numbers of VP1 gene of *Parvovirus*, and viral sequences extracted from other species genomes. **B** Cartoon and collapsed tree showing the relation and number of Clades

PRDs are also present in other, non-galling aphids. We performed a detailed phylogenetic analysis of *S. chinensis* PRDs with other aphids. We did NCBI BLAST searches using *S. chinensis* PRDs as queries against genomes of other aphids and downloaded the genomes with the best hits. We also downloaded parvovirus coat protein VP1 genes and performed phylogenetic analyses to investigate the origin of *S. chinensis* PRDs. The results indicated that one clade of a total of 13 PRDs from the *S. chinensis* genome grouped with sequences extracted from other aphids i.e., *Myzus persicae*, *Acyrthosiphon pisum*, and *Sipha flava*, suggesting common ancestry (Fig. 7a). While one PRD (SchiG000450.1) nested with the single cell protist *Abeoforma* *Parvovirus* coat protein VP1 (Accession BK010894.1) in a clade containing *Parvovirus* VP1 sequences of *Semian* *Parvovirus*, *Myzus percicae*, African termite* Densovirus*, and *Solenopsis invicta*. The remaining PRDs clustered separately into five different clades, and a single branch of one PRD, which indicated multiple and independent integrations of these sequences in the *S. chinensis* genome (Fig. 7A and B). Overall, all the PRDs identified in *S. chinensis* genomes belong to seven lineages (Fig. 7B). The exact role and function of these endogenized genes are unknown in these aphids. However, a recent study reported horizontally transferred *Parvovirus* non-structural genes in pea aphid genomes to have roles in wing plasticity in response to crowding [79]. Here, for the first time, we present the integration and endogenization of *Parvovirus*-related DNA sequences (PRDs) in a galling aphid genome. Further studies and research will be required to investigate the role and functions of these endogenized sequences in this and other aphids.

### Cytochrome P450 genes and gall-inducing genes of *S. chinensis*

Like all other aphids, *S. chinensis* feeds on the sap of its host plant, and in so doing it must detoxify any toxic plant metabolites that are present. Within the genome of *S. chinensis*, we have identified 36 cytochrome P450 genes, named ScP450s, constituting approximately 0.21% of the total genes. Among these, 35 genes appear to be functional, while one is likely a pseudogene due to transposable elements insertions in it [80]. We employed the standard nomenclature from the Cytochrome P450 homepage [81] to name and classify these 36 genes. They have been classified into four clans, 16 families, and 18 subfamilies, with the most prominent families being CYP6, encompassing ten genes, and CYP4, which includes eight genes (Additional file 1: Table S11). Comparatively, the number of P450 genes was fewer than in all the ten aphid genomes downloaded from GenBank (Additional file 1: Table S9). Compared to other insects, especially the ten aphids included in this study, the number of P450s in *S. chinensis* is fewer, i.e., 48 in *D*. *noxia*, 58 in *A*. *glycine*, 75 in *R*. *maidis*, 83 in *A*. *pisum*, 66 in *A*. *gossypii*, and 115 in *M*. *persicae*. The fewer P450s genes in *S. chinensis* compared to other aphids might be related to *S. chinensis* being an oligophagous insect with only two definitive hosts, i.e., the sole primary host, *Rhus chinensis*, and secondary hosts comprising only three moss species from the same family. In contrast, most of the other aphids are polyphagous, with many host plants requiring more P450 genes to detoxify the metabolites of more diverse host plants [82]. All the P450 genes identified in *S. chinensis* were extracted from the genome (Additional file 3), and their position on chromosomes was also located (Fig. 6, Additional file 1: Table S11)

The average length of ScP450s with complete open reading frames was 498 amino acids (aa), which is consistent with the average P450 gene length in other insects [83]. The 36 ScP450s were located on ten chromosomes, in which chromosome 1 contained the highest number of nine, and chromosome 6 had 8 ScP450s. Almost half of the genes were present as a cluster of two or more on each of the six chromosomes, while the remaining were present individually. We did not find any duplicated ScP450s, suggesting the absence of any duplication event during evolution.

**Fig. 8:**
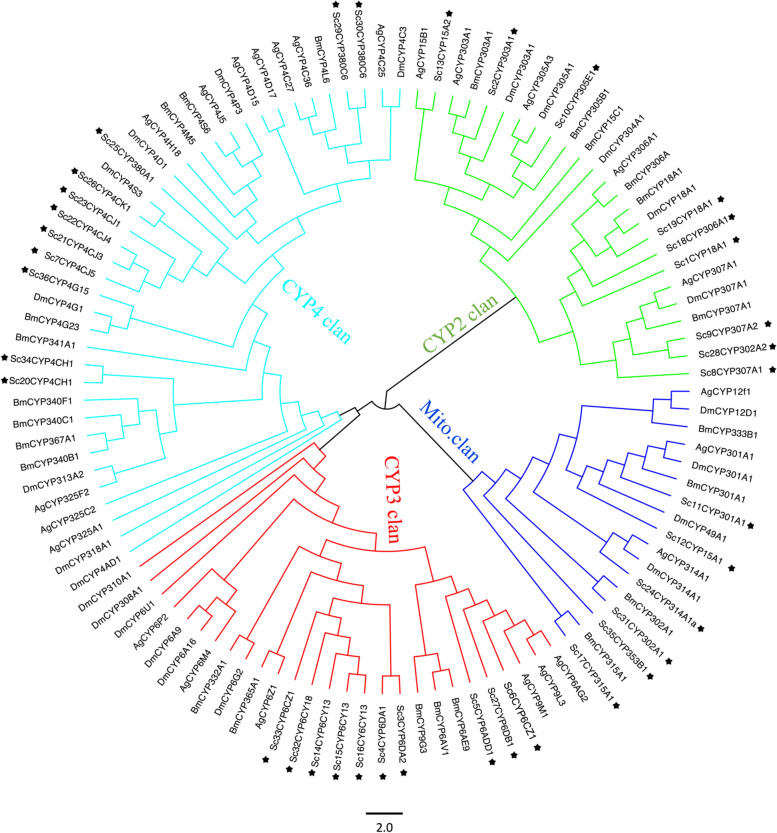
Phylogenetic relationships of the P450s of *Schlechtendalia chinensis*, *Anopheles gambiae*, *Bombyx mori*, and *Drosophila melanogaster*. The four P450 clans are represented by different colored branches. Red, blue, cyan, and green branches represent clans of the CYP3, mitochondrial, CYP4, and CYP2, respectively. The P450s of *S. chinensis*, *A. gambiae*, *B. mori*, and *D. melanogaster* P450s are labeled as ScCYP, AgCYP, BmCYP, and DmCYP, respectively with the first two letters representing the acronym of their scientific names, while the next three letter followed by digits indicates the family name, and then followed by subfamily (See Additional Table 11). The peripheral black stars indicate the P450s of *S. chinensis*

A maximum likelihood (ML) phylogenetic tree of the P450 superfamily was constructed using three other insect species (*Anopheles gambiae*, *Drosophila melanogaster*, and *Bombyx mori*) with already known P450 classifications to identify gene orthologs (Fig. 8). The phylogenetic tree resolved the four expected insect P450 groups of the CYP2, CYP3, CYP4, and mitochondrial clans. The CYP3 clan contained a single family CPY6 with less evolutionary divergence as compared to the other clan. The mitochondrial clan contained the fewest ScP450 genes, but all six belonged to different families. Although the BLASTn searches showed the closest relationship of all ScP450s with *Acyrthosiphon pisum* P450 sequences, they were not included in the analysis due to the absence of a complete dataset. The CYP4 clan contained 11 ScP450 genes, followed by CYP3 and CYP2, which contained 10 and 9 ScP450 genes, respectively (Fig. 8, Additional Table 11).

Gall formation is a defensive response of some plants to galling microorganisms and insects. Insects in several different orders can induce galls on their hosts, but the exact mechanisms by which galls are induced and developed are still uncertain. Some studies have shown that gall induction is species-specific, and galling insects induce galls on their host by delivering effector proteins into plant tissues through their saliva during feeding [84–86]. Saliva injection by *S. chinensis* in the leaf cells of the host plant induces the formation of the gall. Recently, LC-MS/MS analysis of *S. chinensis* saliva identified 31 *S. chinensis* proteins, some of which may conceivably play a role in gall induction [87]. The genes coding for these specific salivary proteins were also annotated and present in our *S. chinensis* complete data set (15,013 genes). The proteins coded by these genes are mostly related to binding activities, including DNA-, ATP-, protein-, and iron-binding.

## Discussion

The superfamily Aphidoidea consists of over 5,000 species and 500 genera; some are important economic pests [88]. While the great majority of species are free-living, galling aphids like the horn-gall aphid *Schlechtendalia chinensis* have evolved unique adaptations to redirect the development of their host plants. The complete genome of *S. chinensis* will provide a rich resource for studies of behavior, host-plant interactions, and life cycles for future genomic and genetic studies [18]. Here, we performed a high-quality chromosomal-level genome assembly of *S. chinensis* by combining different sequencing strategies, which yielded thirteen chromosome-level scaffolds and over 15,000 genes. Recently, a chromosome level genome assembly of *S. chinensis* was published, highlighting the interaction of this aphid with its host plant [9]. However, our genome assembly provides more in-depth information about several key aspects, for example Cytochrome P450 gene family, Endogenization of PRDs, and genome duplication events. Furthermore, our genome assembly yielded a larger genome size of 344.59 Mb and predicted a greater number of genes (15,013) compared to the previous study, which reported a size of 271.52 Mb and 14,089 genes [9]. This genome assembly of *S. chinensis* will further improve information about this species and provide valuable insights for comparative genome studies of galling aphids with other aphids. The exact mechanism and gall induction by this insect are still poorly understood, however, recent studies have reported several genes encoding salivary glands proteins that are expressed differentially during *S. chinensis* gall induction and may play a role in gall formation [9, 87]. The present study further adds more genomic information and data about *S. chinensis* that could be used in future studies to understand the underlying mechanism of gall insects’ interaction with their host plant.

*Parvoviruses* cause a wide range of diseases in insects and other arthropods; however, the persistence mechanism of their infection is unknown [89]. Integration of viral genome sequences belonging to the family *Parvoviridae* has been reported in arthropods, including the aphids *Myzus persicae* and *A. pisum* [74]. The endogenization of *Parvoviruses* in the genomes of *M. persicae* from different geographical origins suggested an old and stable integration of these viruses [76]. We also found many *Parvovirus*-like DNA sequences (PRDs) integrated into the genome of *S. chinensis*, which mainly were related to structural coat protein VP1. However, while some studies detected both structural and non-structural genes integrated into the host genome [74], sequences related to non-structural genes were not detected in our study. While the integration mechanism of *Parvoviruses* remains unknown in at least one aphid (*M. persicae*) [76], the high capacity of *Parvoviruses* to incorporate into the host genome is probably due to their replication cycle in the nucleus [90, 91].

The coding capacity retained in PRDs with amino acid sequences ranging from 130 to 660 as yet have unknown function, while others were related to phospholipase A2 (PLA2) activity (GO and KEGG analysis) and may have a role in viral infection [92]. Previous studies on aphids also reported that the *Parvovirus* integrated sequences remain potentially active [76]. A recent study provided experimental evidence of the horizontal transfer of *Parvovirus* non-structural genes into the genome of apple aphids [79]. These genes were shown to play a role in wing plasticity, triggering the development of wings in response to crowding in aphids [79]. As such, these integrated PRDs appear to have a role in aphid adaptation to environmental changes.

The phylogenetic analysis of PRDs showed that only two PRDs in *S. chinensis* were clustered with other aphids, indicating their common origin. Because all other PRDs clustered into five different clades, we interpret this to reflect at least five independent integrations of different strains of *parvoviruses* in the genome. *Parvoviruses* integration has been reported in many animals and arthropods, including aphids. Our current study of the *S. chinensis* genome reveals novel endogenization of PRDs for the first time in a galling aphid. Further investigation is necessary to analyze their role and possible contributions to the genomic evolution of horn-gall aphids.

The cytochrome P450 gene family plays a crucial role in the resistance and adaptation of aphids to plant chemical metabolites [93], and an analysis of P450s at the genomic level has provided information on the metabolic functions of their expressed proteins [94]. Previous studies suggested that the greater hydrolytic activity of P450s in insects conferred resistance to insecticides [95, 96]. As the saliva of *S. chinensis* also contained P450 proteins which could be related to gall induction in their host [87], we focused on identifying P450 genes in the *S. chinensis* genome. The P450 genes we identified were distributed among multiple chromosomes and were classified into the expected four clans to which all the insect P450s belong [81]. The fewer P450 genes in *S. chinensis* as compared to other aphids might be related to its narrow host-plant relationships, as *S. chinensis* is specialist, feeding on only *Rhus chinensis* and moss for its host-alternating life cycle. In contrast, several of the other non-galling aphids are polyphagous, having many hosts from different genera. Because cytochrome P450 gene expression is targeted towards countering complex environmental challenges [97], polyphagous insects may have more P450 genes as a response to selection by their more diverse diet. Limited exposure to a narrow range of host plants might have led to genomic streamlining in *S. chinensis*, favoring a smaller set of functional genes tailored specifically for metabolizing the chemicals present in its specialized host plants. This reduction in genetic diversity might reflect functional optimization for the encountered plant compounds. A significant correlation has been observed between the number of P450 genes and the range of hosts in the non-galling specialist aphids *A. pisum* (83 P450s) and generalist *M. persicae* (115 P450s) in the previous studies [98]. Due to its wider host ranges (more than a hundred species from 40 families), the species *M. persicae* exhibits a 40% higher number of P450 genes than *A. pisum* that feeds exclusively in the family Fabaceae [98]. As mentioned above, the species *S. chinensis* has only two very specific alternative host plants, which results in limited ranges of host metabolites exposure and a lower number of P450s. Our result together with the previous finding supports the notion that narrow diversity in host plants might impact the genetic composition, gene family evolution, and metabolic adaptation in aphids.

Like all other aphids, galling aphids produce saliva containing different enzymes and proteins, which help them probe and feed on host plants. Previous studies have identified salivary and salivary gland proteins in *S. chinensis* and other free-living aphids [87]. Our annotation of the *S. chinensis* genome serves as a source of information for establishing the genetic and genomic basis of these proteins, some of which could play a role in gall formation. Cytochrome P450 monooxygenase and cytochrome b5-like heme/steroid binding domain, which was identified in *S. chinensis* saliva previously [87], were also identified and annotated in our study.

In summary, we provide a high-quality chromosome-level genome assembly of *S. chinensis*, which can serve as a reference genome for gall-forming aphids. It will be valuable for future comparative genomic studies of aphids and other phloem-feeding insects, especially gall-inducing insects. This study also provides insights into the endogenization of *Parvoviruses* in aphid genomes; such viruses might play essential roles in *S*. *chinensis* physiology and the biology and evolution of aphids in general. The chromosome-level genome assembly will facilitate future studies on the adaptations involved in gall formation in *S. chinensis* and support the development of sustainable strategies for cultivating gall-forming insects on a commercial scale.

### Supplementary Information

**Additional file 1: Figure S1. **Distribution frequency and coverage of 19-mers in *Schlechtendalia chinensis* genome. **Figure S2.** The number of genes integrated by EVM that are supported by the three prediction methods is counted separately, as shown in the figure, it can be seen that most of the genes are derived from the transcriptome and homologous predictions, indicating that the prediction quality is high. **Figure S3.** Functional classification of all the genes predicted in the genome of *S. chinensis*. **Figure S4.** Functional classification of *S. chinensis* specific gene family by GO [33] and KEGG [34] enrichment analysis using clusterProfile v3.14.0. **Figure S5.** Functional classification of expanded gene families in *S. chinensis* genome by GO and KEGG [33] enrichment analysis using clusterProfile v3.14.0. **Figure S6.** Functional classification of contracted gene families in *S. chinensis* genome by GO and KEGG [34] enrichment analysis using clusterProfile v3.14.0. **Figure S8.** Functional classification of positively selected gene families in *S. chinensis* genome by KEGG [34] enrichment analysis. **Table S1.** Detail statistics of PacBio library raw data and clean data. **Table S2.** Statistics of the clean sequence reads produced from PacBio library. **Table S3.** Statistics of all the scaffolds anchored on 13 chromosomes and their order length and numbers. **Table S4.** Detailed information about the contigs and scaffolds of the sequenced genome. **Table S5.** BUSCO analysis result against data set of 939 metazoan species. **Table S6.** Statistics of Repeat elements in *S. chinensis* genome. **Table S7.** Prediction of total number of genes in the genome of *Schlechtendalia chinensis* by the combinations of different predicting pipelines and software. **Table S8.** Number of genes annotated and information of pipelines used for the annotation of genes in *Schlechtendalia chinensis*.** Table S9.** Species name and genome deposited database of aphids used in comparative analysis. **Table S10.** Table showing positively selected genes ID, p value and selected sites. Also shows their respective gene families. **Table S11.** Table shows classification, nomenclature and the number of exons in each gene, along with their location on chromosomes. 

**Additional file 2. **Annotation information of *Parvovirus* like DNA sequences.

**Additional file 3. **Sequence Data of Cytochrome P450 gene family.

## Data Availability

High-throughput sequencing data analysed in this project and the whole Genome project (including assembly and annotation) are deposited under BioProject (PRJNA833747) and BioSample (SAMN28016330) to NCBI GenBank. Individual accession number for each chromosome scaffold will be updated later when received from NCBI. While all other data generated during this study are included in this article and its Additional files.
