# Supplementary material for: A chromosome-level genome assembly of the Rhus gall aphid Schlechtendalia chinensis provides insight into the endogenization of Parvovirus-like DNA sequences
Source: BMC Genomics. 2024 Jan 2;25:16. doi: 10.1186/s12864-023-09916-y (PMC10759679; doi:10.1186/s12864-023-09916-y)
Supplement: Supplementary file 1 — Additional file 1: Figure S1. Distribution frequency and coverage of 19-mers in Schlechtendalia chinensis genome. Figure S2. The number of genes integrated by EVM that are supported by the three prediction methods is counted separately, as shown in the figure, it can be seen that most of the genes are derived from the transcriptome and homologous predictions, indicating that the prediction quality is high. Figure S3. Functional classification of all the genes predicted in the genome of S. chinensis. Figure S4. Functional classification of S. chinensis specific gene family by GO [33] and KEGG [34] enrichment analysis using clusterProfile v3.14.0. Figure S5. Functional classification of expanded gene families in S. chinensis genome by GO and KEGG [33] enrichment analysis using clusterProfile v3.14.0. Figure S6. Functional classification of contracted gene families in S. chinensis genome by GO and KEGG [34] enrichment analysis using clusterProfile v3.14.0. Figure S8. Functional classification of positively selected gene families in S. chinensis genome by KEGG [34] enrichment analysis. Table S1. Detail statistics of PacBio library raw data and clean data. Table S2. Statistics of the clean sequence reads produced from PacBio library. Table S3. Statistics of all the scaffolds anchored on 13 chromosomes and their order length and numbers. Table S4. Detailed information about the contigs and scaffolds of the sequenced genome. Table S5. BUSCO analysis result against data set of 939 metazoan species. Table S6. Statistics of Repeat elements in S. chinensis genome. Table S7. Prediction of total number of genes in the genome of Schlechtendalia chinensis by the combinations of different predicting pipelines and software. Table S8. Number of genes annotated and information of pipelines used for the annotation of genes in Schlechtendalia chinensis. Table S9. Species name and genome deposited database of aphids used in comparative analysis. Table S10. Table showing positively select [file 12864_2023_9916_MOESM1_ESM.pdf]

Supplementary file 1

A chromosome-level genome assembly of the *Rhus* gall aphid *Schlechtendalia chinensis* provides insight into the endogenization of *Parvovirus*-like DNA sequences

Aftab Ahmad[1], Carol D. von Dohlen[2], Zhumei Ren[1*]

[1]School of Life Science, Shanxi University, Taiyuan, Shanxi, China

[2]Department of Biology, Utah State University, Logan, Utah, United States of America

[*]Correspondence author: Zhumei Ren

Email: zmren@sxu.edu.cn

Supplementary Figures

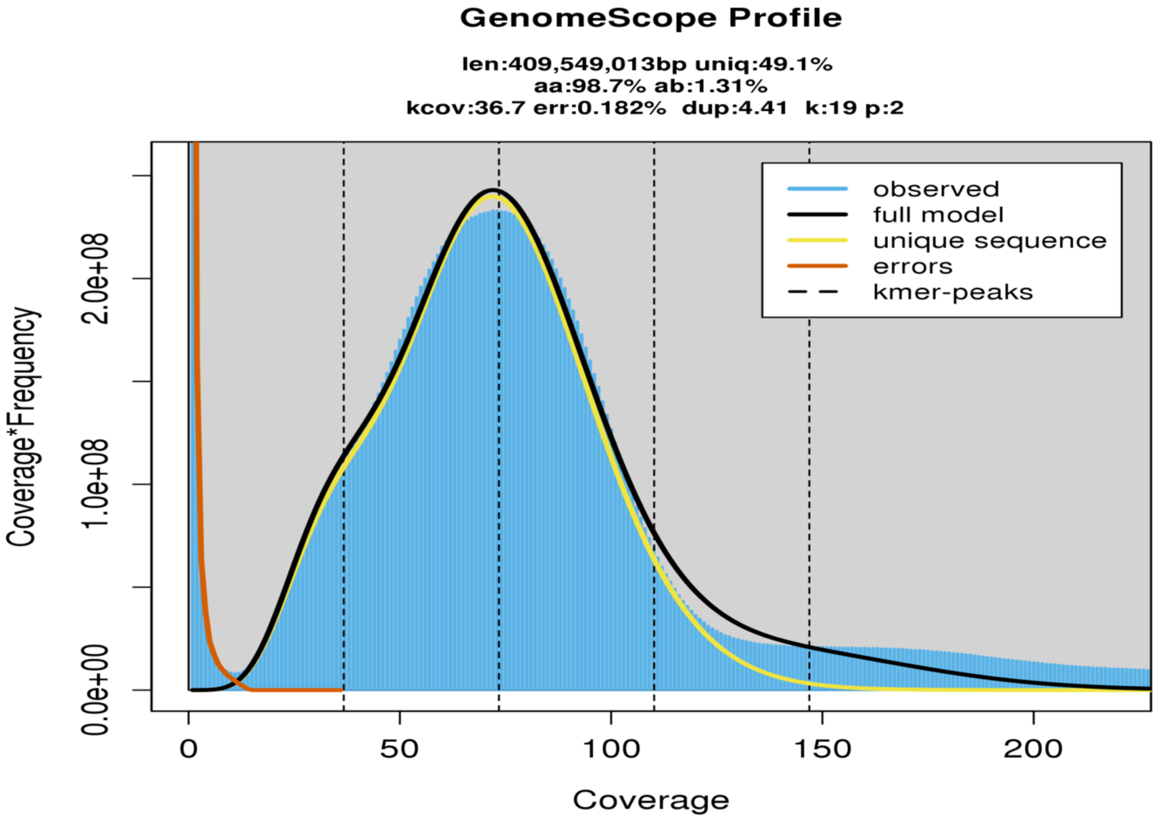

Figure S1. Distribution frequency and coverage of 19-mers in Schlechtendalia chinensis genome.

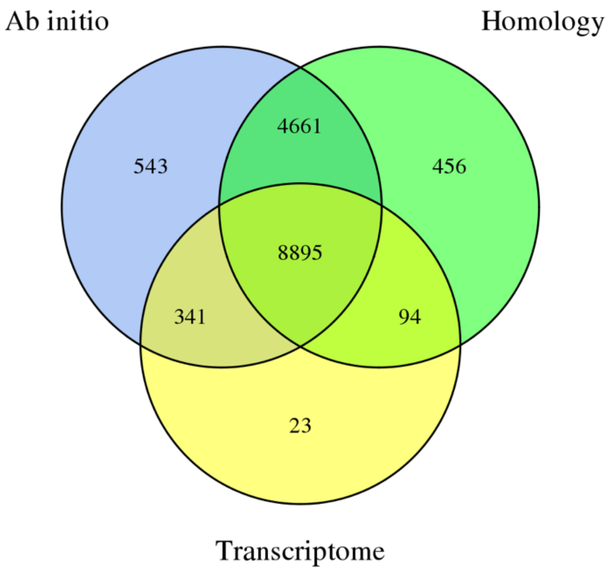

Figure S2. The number of genes integrated by EVM that are supported by the three prediction methods is counted separately, as shown in the figure, it can be seen that most of the genes are derived from the transcriptome and homologous predictions, indicating that the prediction quality is high.

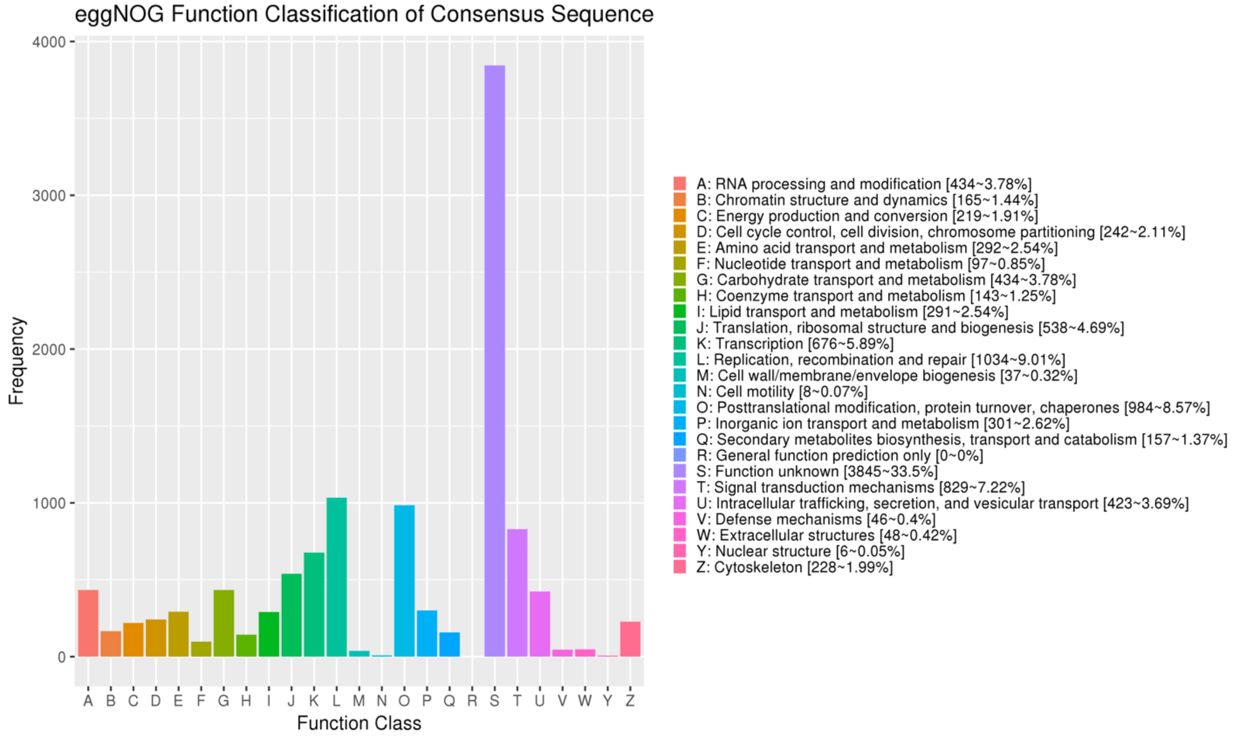

Figure S3. Functional classification of all the genes predicted in the genome of *S. chinensis*.

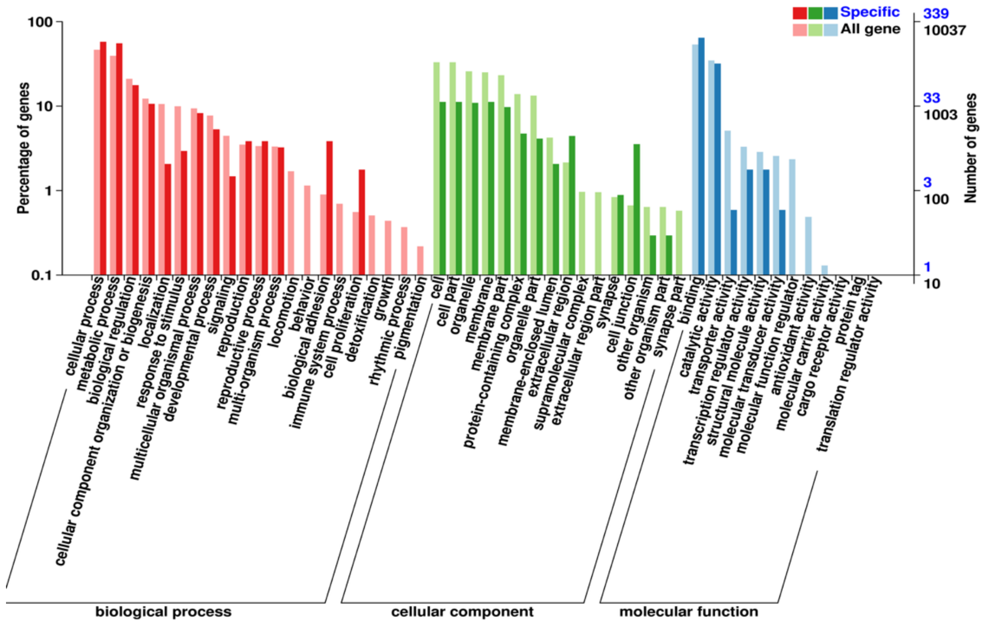

Figure S4. Functional classification of *S. chinensis* specific gene family by GO [33] and KEGG [34] enrichment analysis using clusterProfile v3.14.0.

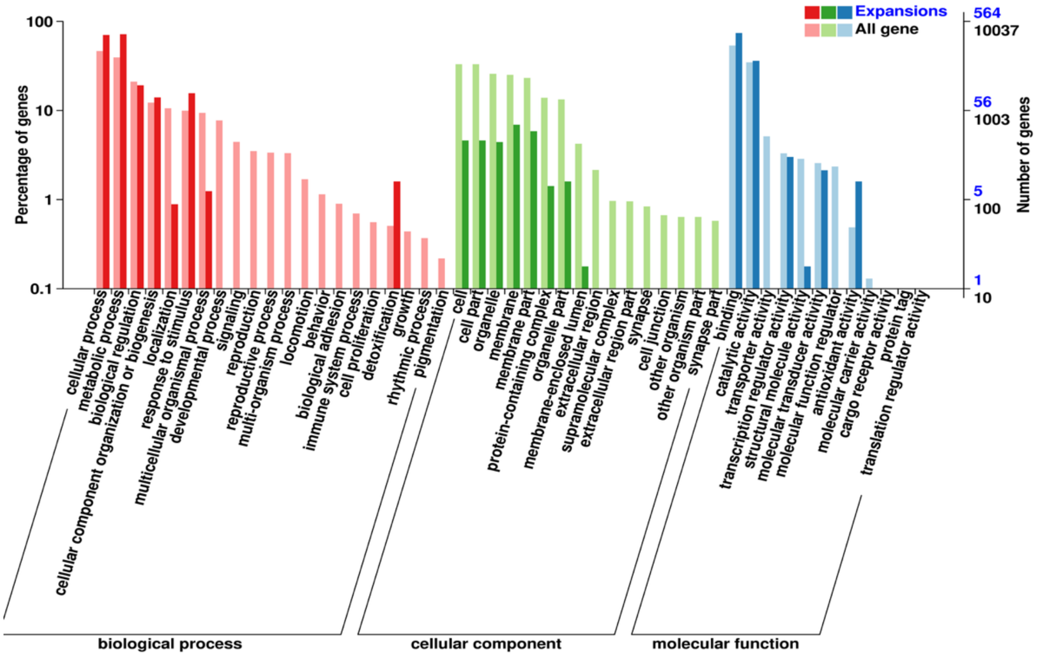

Figure S5. Functional classification of expanded gene families in *S. chinensis* genome by GO and KEGG [33] enrichment analysis using clusterProfile v3.14.0.

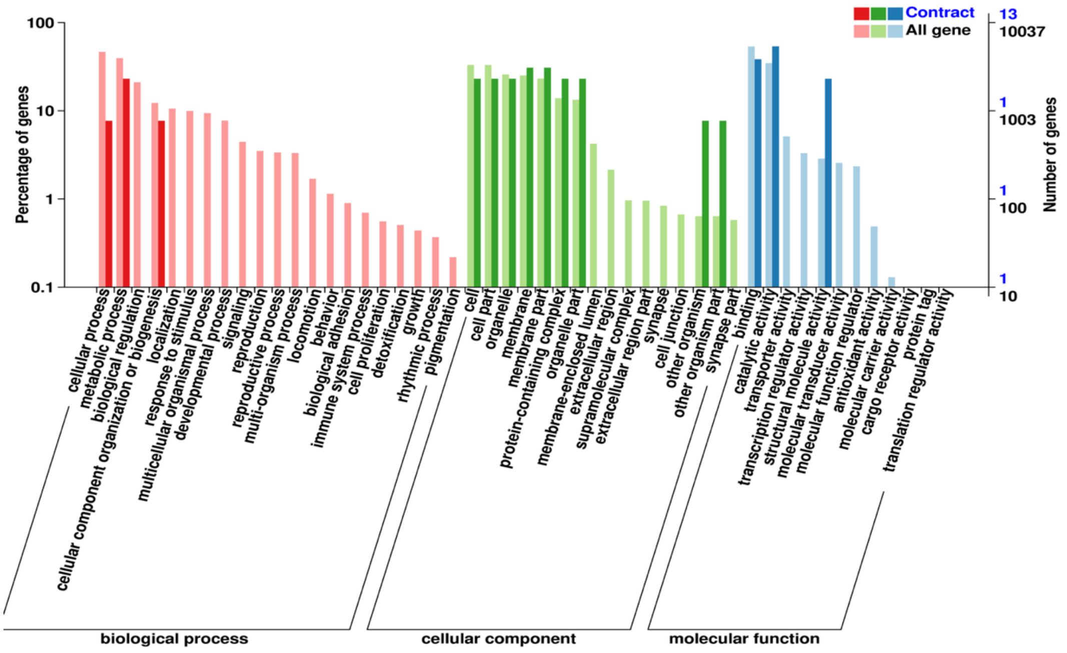

Figure S6. Functional classification of contracted gene families in *S. chinensis* genome by GO and KEGG [34] enrichment analysis using clusterProfile v3.14.0.

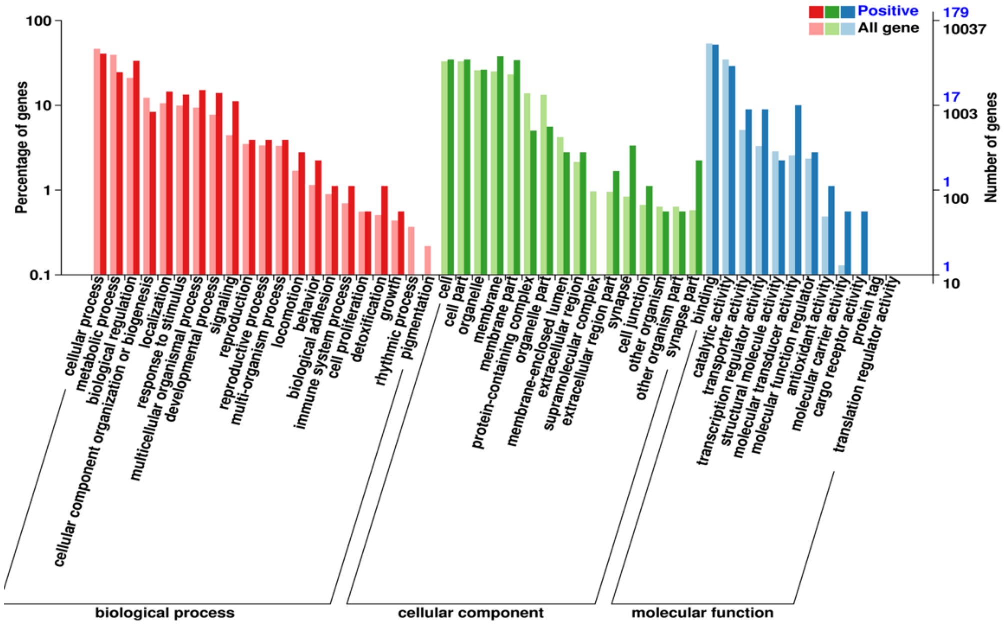

Figure S7. Functional classification of positively selected gene families in *S. chinensis* genome by GO [33] enrichment analysis using clusterProfile v3.14.0.

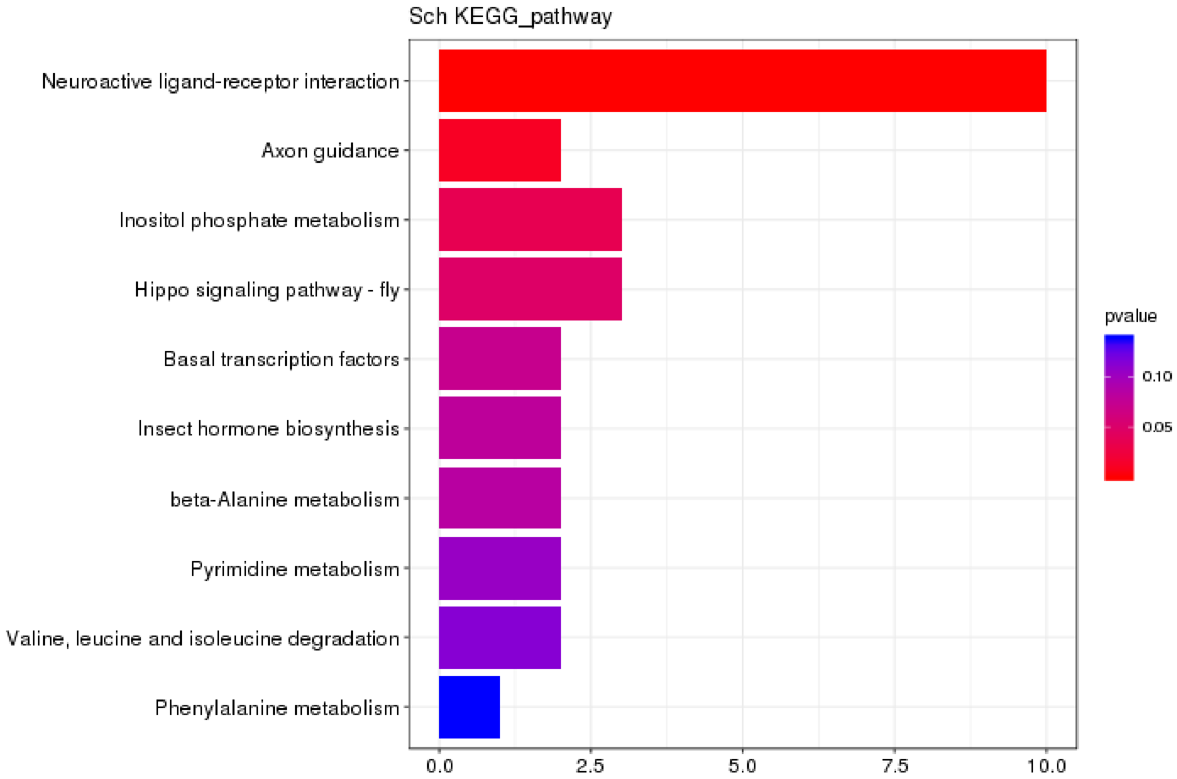

Figure S8. Functional classification of positively selected gene families in *S. chinensis* genome by KEGG [34] enrichment analysis.

Supplementary Tables

Table S1. Detail statistics of PacBio library raw data and clean data.

PacBio library	Reads No.	Total Bases (bp)	Reads N50 (bp)	Mean Length (bp)	Longest Read (bp)
**Raw data**	35,110,707	510,486,774,013	16,407	14,539	451,103
**Clean data**	2,257,809	36,046,261,292	16,203	15,965	45,972

Table S2. Statistics of the clean sequence reads produced from PacBio library.

Length	Number	Total length	Average length
500~2000	415	616,841	1,486.36
2000~4000	501	1,410,039	2,814.45
4000~6000	372	1,867,191	5,019.33
6000~8000	837	6,021,634	7,194.31
8000~10000	1,765	15,908,570	9,013.35
10000~12000	25,874	299,983,495	11,594.01
12000~14000	322,829	4,259,197,478	13,193.35
14000~16000	782,171	11,822,455,384	15,114.92
16000~18000	805,054	13,600,727,346	16,894.18
18000~	317,991	6,038,073,314	18,988.19

Table S3. Statistics of all the scaffolds anchored on 13 chromosomes and their order length and numbers.

Chromosomes no.	Clustered Scaffolds	Cluster length	Order length	Order number
Chr.1	11	123,018,421	8	122,785,658
Chr.2	1	22,033,345	1	22,033,345
Chr.3	1	21,282,667	1	21,282,667
Chr.4	1	21,091,861	1	21,091,861
Chr.5	2	20,997,589	1	20,977,384
Chr.6	1	20,798,509	1	20,798,509
Chr.7	1	15,397,934	1	15,397,934
Chr.8	1	13,329,565	1	13,329,565
Chr.9	5	12,370,492	1	12,217,479
Chr.10	2	12,226,705	1	12,191,903
Chr.11	1	11,734,047	1	11,734,047
Chr.12	1	11,250,006	1	11,250,006
Chr.13	1	10,465,550	1	10,465,550
Total anchored	29	315,996,691	20	315555908
Unanchored	160			29,013,330

Table S4. Detailed information about the contigs and scaffolds of the sequenced genome.

Contigs no.	CtgLength	CtgN50	CtgN90	CtgMax	GapNum	MaxGap	GapLen
196	344,569,235	20,977,384	2992,478	103,005,580	7	1100	700
Scaffolds no.	Scf-Length	ScfN50	ScfN90	ScfMax	Scf gap	MaxGap	Gaplength
189	344,569,235	21,091,861	10,465,550	122,786,358	0	0	0

Table S5. BUSCO analysis result against data set of 939 metazoan species.

Complete BUSCO	Complete and single copy	Complete and duplicated	Fragmented	Missing BUSCO	Total lineage
900 (94.34%)	879(92.14%)	21 (2.20)	7 (0.73%)	47 (4.93)	954

Table S6. Statistics of Repeat elements in *S. chinensis* genome.

**Repeats type**	**Number**	**Length**	**Rate (%)**
Transposons			
Class I: Retroelements	96,053	33,038,399	9.59
Class II: DNA transposons	212,638	56096,664	16.45
Total	308,691	89,735,022	26.04
Tandem Repeats			
Microsattelites (1-9bp)	344,854	6,021,432	1.75
Mininsatellite (10-99bp)	37,817	2,287,935	0.66
Satellite (≥100bp)	4054	31,259,608	9.07
Total	386,725	39,568,975	11.48

Table S7. Prediction of total number of genes in the genome of *Schlechtendalia chinensis* by the combinations of different predicting pipelines and software.

**Method**	**Software**	**Species**	**Number of genes**
Ab initio	Augustus	-	10,225
	SNAP	-	65,225
Homology	GeMoMa	*A. pisum*	14,076
		C. cedri	15,760
		D. melanogaster	7,099
		S. flava	13,695
RNAseq	GeneMarkS-T	-	9,635
	PASA	-	6,296
Integration	EVM	-	15,013

Table S8. Number of genes annotated and information of pipelines used for the annotation of genes in *Schlechtendalia chinensis.*

Annotation database	Number of genes Annotated	Annotation Ratio
GO Annotation	10,037	66.86
KEGG Annotation	10,997	73.25
KOG Annotation	7,762	51.7
Pfam Annotation	11,447	76.25
Swissport Annotation	9,847	65.59
TrEMBL Annotation	14,546	96.89
eggNOG Annotation	10,795	71.9
Nr Annotation	14,238	94.84
All Annotated	14,582	97.13

Table S9. Species name and genome deposited database of aphids used in comparative analysis.

Species	Download
Sipha flava	https://ftp.ncbi.nlm.nih.gov/genomes/all/GCF/003/268/045/GCF_003268045.1_YSA_version1/
Bemisia tabaci	https://ftp.ncbi.nlm.nih.gov/genomes/all/GCF/001/854/935/GCF_001854935.1_ASM185493v1/
Aphis glycine	https://ftp.ncbi.nlm.nih.gov/genomes/all/GCA/009/761/285/GCA_009761285.1_PRSTRT_AglyBT1_v1/
Myzus persicae	https://ftp.ncbi.nlm.nih.gov/genomes/all/GCF/001/856/785/GCF_001856785.1_MPER_G0061.0/
Aphis gossypii	https://www.ncbi.nlm.nih.gov/genome/?term=cotton+aphid
A.cyrthosiphon pisum	https://ftp.ncbi.nlm.nih.gov/genomes/all/GCF/005/508/785/GCF_005508785.1_pea_aphid_22Mar2018_4r6ur/
Cinara cedri	https://denovo.cnag.cat/ccedri_data?fid=513#block-likable-page-title
Diuraphis noxia	https://ftp.ncbi.nlm.nih.gov/genomes/all/GCF/001/186/385/GCF_001186385.1_Dnoxia_1.0/
Melanaphis sacchari	https://ftp.ncbi.nlm.nih.gov/genomes/all/GCF/002/803/265/GCF_002803265.2_SCAv2.0/
Aphis craccivora	https://ftp.ncbi.nlm.nih.gov/genomes/all/GCA/009/835/225/GCA_009835225.1_LBME_Acra_1.0/
Rhopalosiphum maidis	https://ftp.ncbi.nlm.nih.gov/genomes/all/GCF/003/676/215/GCF_003676215.2_ASM367621v3/

Table S10. Table showing positively selected genes ID, p value and selected sites. Also shows their respective gene families.

Gene ID	Branch p value	Sites	Gene family
Schi05G001030	0.000000852	455, S,0.958*	OG0000617
Schi03G004090	0.016935928	8, E,0.963*	OG0001178
Schi02G003940	0.000019853	54, S,0.964*	OG0001189
Schi01G007870	0.011928664	53, A,0.962* 83,Q,0.957* 87,D,0.971* 154,A,0.981* 157,V,0.983* 169,S,0.974*	OG0001465
Schi12G001200	0.000578590	4, G,0.976* 82,D,0.982* 378,R,0.965* 545,N,0.986*	OG0001514
Schi01G021000	0.001263182	214, S,0.952* 217,N,0.952* 219,T,0.970* 220,S,0.970*	OG0001624
Schi01G013780	0.000000576	357, E,0.959* 367,R,0.972*	OG0001652
Schi06G003430	0.000000054	779, S,0.960*	OG0001708
Schi05G007890	0.038969694	20, S,0.956*	OG0001734

Table S11. Table shows classification, nomenclature and the number of exons in each gene, along with their location on chromosomes.

Gene ID	Gene name	P450 clan	Family	Subfamily	No. of exons	Location
Schi01G035210	Sc7CYP4CJ5	CYP4	CYP4	CJ	9	Chr.1
Schi06G008170	Sc20CYP4CH1	-	CYP4	CH	8	Chr.6
Schi06G010000	Sc21CYP4CJ3	-	CPY4	CJ	11	Chr.6
Schi06G010010	Sc22CYP4CJ4	-	CYP4	CJ	12	Chr.6
Schi06G010020	Sc23CYP4CJ1	-	CYP4	CJ	11	Chr.6
Schi08G001700	Sc25CYP380A1	-	CYP380	A	10	Chr.8
Schi08G002470	Sc26CYP4CJ4	-	CYP4	CK	10	Chr.8
Schi09G000180	Sc29CYP380C6	-	CYP380	C	12	Chr.9
Schi09G000200	Sc30CYP380C6	-	CYP380	C	12	Chr.9
Schi10G003170	Sc34CYP4CH1	-	CYP4	CH	5	Chr.11
Schi12G001970	Sc36CYP4G15	-	CYP4	G	12	Chr.12
Sch01G019250	Sc3CYP6DA2	CYP3	CYP6	DA	5	Chr.1
Sch01G019260	Sc4CYP6DA1	-	CYP6	AD	5	Chr.1
Sch01G031590	Sc5CYP6ADD1	-	CYP6	ADD	6	Chr.1
Schi01G033130	Sc6CYP6CZ1	-	CYP6	CZ	2	Chr.1
Schi05G007440	Sc14CYP6CY13	-	CYP6	CY	4	Chr.5
Schi05G007430	Sc15CYP6CY13	-	CYP6	CY	4	Chr.5
Schi05G007400	Sc16CYP6CY13	-	CYP6	CY	4	Chr.5
Schi08G004540	Sc27CYP6CY13	-	CYP6	DB	7	Chr.8
Schi10G003160	Sc32CYP6CY18	-	CYP6	CY	4	Chr.10
Schi10G003170	Sc33CYP6CZ1	-	CYP6	CZ	5	Chr.10
Schi01G051540	Sc1CYP18A1	CYP2	CYP18	A	-	Chr.1
Schi01G003300	Sc2CYP303A1	-	CYP303	A	7	Chr.1
Schi01G040830	Sc8CYP307A1	-	CYP307	A	3	Chr.1
Schi01G040920	Sc9CYP307A2	-	CYP307	A	3	Chr.1
Schi02G004050	Sc10CYP305E1	-	CYP305	E	8	Chr.2
Schi04G005520	Sc13CYP15A2	-	CYP15	A	8	Chr.4
Schi06G003040	Sc18CYP306A1	-	CYP306	A	6	Chr.6

Schi06G003050	Sc19CYP306A1	-	CYP18	A	5	Chr.6
Schi09G044340	Sc28CYP302A2	-	CYP302	A	3	Chr.1
Schi04G003130	Sc11CYP306A1	Mitochondrial	CYP301	A	10	Chr.4
Schi04G003120	Sc12CYP306A1	-	CYP15	A	10	Chr.4
Schi06G002630	Sc17CYP315A1	-	CYP315	A	8	Chr.6
Schi06G010840	Sc24CYP314A1a	-	CYP314	A	10	Chr.6
Schi10G000210	Sc31CYP302A1	-	CYP302	A	9	Chr.10
Schi11G001010	Sc35CYP353A1	-	CYP353	B	8	Chr.11
